# Genomic Predictors for Recurrence Patterns of Hepatocellular Carcinoma: Model Derivation and Validation

**DOI:** 10.1371/journal.pmed.1001770

**Published:** 2014-12-23

**Authors:** Ji Hoon Kim, Bo Hwa Sohn, Hyun-Sung Lee, Sang-Bae Kim, Jeong Eun Yoo, Yun-Yong Park, Woojin Jeong, Sung Sook Lee, Eun Sung Park, Ahmed Kaseb, Baek Hui Kim, Wan Bae Kim, Jong Eun Yeon, Kwan Soo Byun, In-Sun Chu, Sung Soo Kim, Xin Wei Wang, Snorri S. Thorgeirsson, John M. Luk, Koo Jeong Kang, Jeonghoon Heo, Young Nyun Park, Ju-Seog Lee

**Affiliations:** 1 Department of Systems Biology, Division of Cancer Medicine, The University of Texas MD Anderson Cancer Center, Houston, Texas, United States of America; 2 Kleberg Center for Molecular Markers, Division of Cancer Medicine, The University of Texas MD Anderson Cancer Center, Houston, Texas, United States of America; 3 Division of Gastroenterology and Hepatology, Department of Internal Medicine, Korea University College of Medicine, Seoul, Korea; 4 Department of Pathology and Brain Korea 21 Project for Medical Science, Yonsei University College of Medicine, Seoul, Korea; 5 ASAN Institute for Life Sciences, Asan Medical Center, Department of Medicine, University of Ulsan College of Medicine, Seoul, Korea; 6 Department of Life Sciences, Division of Life and Pharmaceutical Sciences, Center for Cell Signaling and Drug Discovery Research, Ewha Womans University, Seoul, Korea; 7 Department of Hematology-Oncology, Inje University Haeundae Paik Hospital, Busan, Korea; 8 Institute for Medical Convergence, Yonsei University College of Medicine, Seoul, Korea; 9 Department of GI Medical Oncology, Division of Cancer Medicine, The University of Texas MD Anderson Cancer Center, Houston, Texas, United States of America; 10 Department of Pathology, Department of Internal Medicine, Korea University College of Medicine, Seoul, Korea; 11 Department of Surgery, Department of Internal Medicine, Korea University College of Medicine, Seoul, Korea; 12 Korean Bioinformation Center, Korea Research Institute of Bioscience and Biotechnology, Daejeon, Korea; 13 Department of Biochemistry and Molecular Biology, Medical Research Center and Biomedical Science Institute, School of Medicine, Kyung Hee University, Seoul, Korea; 14 Laboratory of Human Carcinogenesis, National Cancer Institute, National Institutes of Health, Bethesda, Maryland, United States of America; 15 Laboratory of Experimental Carcinogenesis, National Cancer Institute, National Institutes of Health, Bethesda, Maryland, United States of America; 16 Department of Pharmacology, National University of Singapore, Singapore; 17 Department of Surgery, Keimyung University School of Medicine, Daegu, Korea; 18 Departments of Molecular Biology and Immunology, Kosin University College of Medicine, Busan, Korea; Harvard Medical School, United States of America

## Abstract

**Background:**

Typically observed at 2 y after surgical resection, late recurrence is a major challenge in the management of hepatocellular carcinoma (HCC). We aimed to develop a genomic predictor that can identify patients at high risk for late recurrence and assess its clinical implications.

**Methods and Findings:**

Systematic analysis of gene expression data from human liver undergoing hepatic injury and regeneration revealed a 233-gene signature that was significantly associated with late recurrence of HCC. Using this signature, we developed a prognostic predictor that can identify patients at high risk of late recurrence, and tested and validated the robustness of the predictor in patients (*n = *396) who underwent surgery between 1990 and 2011 at four centers (210 recurrences during a median of 3.7 y of follow-up). In multivariate analysis, this signature was the strongest risk factor for late recurrence (hazard ratio, 2.2; 95% confidence interval, 1.3–3.7; *p* = 0.002). In contrast, our previously developed tumor-derived 65-gene risk score was significantly associated with early recurrence (*p* = 0.005) but not with late recurrence (*p* = 0.7). In multivariate analysis, the 65-gene risk score was the strongest risk factor for very early recurrence (<1 y after surgical resection) (hazard ratio, 1.7; 95% confidence interval, 1.1–2.6; *p* = 0.01). The potential significance of *STAT3* activation in late recurrence was predicted by gene network analysis and validated later. We also developed and validated 4- and 20-gene predictors from the full 233-gene predictor. The main limitation of the study is that most of the patients in our study were hepatitis B virus–positive. Further investigations are needed to test our prediction models in patients with different etiologies of HCC, such as hepatitis C virus.

**Conclusions:**

Two independently developed predictors reflected well the differences between early and late recurrence of HCC at the molecular level and provided new biomarkers for risk stratification.

*Please see later in the article for the Editors' Summary*

## Abstract 2

In this study, Lee and colleagues develop a genomic predictor that can identify patients at high risk for late recurrence of hepatocellular carcinoma (HCC) and provided new biomarkers for risk stratification.

## Editors' Summary

**Background:**

Primary liver cancer—a tumor that starts when a liver cell acquires genetic changes that allow it to grow uncontrollably—is the second-leading cause of cancer-related deaths worldwide, killing more than 600,000 people annually. If hepatocellular cancer (HCC; the most common type of liver cancer) is diagnosed in its early stages, it can be treated by surgically removing part of the liver (resection), by liver transplantation, or by local ablation, which uses an electric current to destroy the cancer cells. Unfortunately, the symptoms of HCC, which include weight loss, tiredness, and jaundice (yellowing of the skin and eyes), are vague and rarely appear until the cancer has spread throughout the liver. Consequently, HCC is rarely diagnosed before the cancer is advanced and untreatable, and has a poor prognosis (likely outcome)—fewer than 5% of patients survive for five or more years after diagnosis. The exact cause of HCC is unclear, but chronic liver (hepatic) injury and inflammation (caused, for example, by infection with hepatitis B virus [HBV] or by alcohol abuse) promote tumor development.

**Why Was This Study Done?:**

Even when it is diagnosed early, HCC has a poor prognosis because it often recurs. Patients treated for HCC can experience two distinct types of tumor recurrence. Early recurrence, which usually happens within the first two years after surgery, arises from the spread of primary cancer cells into the surrounding liver that left behind during surgery. Late recurrence, which typically happens more than two years after surgery, involves the development of completely new tumors and seems to be the result of chronic liver damage. Because early and late recurrence have different clinical courses, it would be useful to be able to predict which patients are at high risk of which type of recurrence. Given that injury, inflammation, and regeneration seem to prime the liver for HCC development, might the gene expression patterns associated with these conditions serve as predictive markers for the identification of patients at risk of late recurrence of HCC? Here, the researchers develop a genomic predictor for the late recurrence of HCC by examining gene expression patterns in tissue samples from livers that were undergoing injury and regeneration.

**What Did the Researchers Do and Find?:**

By comparing gene expression data obtained from liver biopsies taken before and after liver transplantation or resection and recorded in the US National Center for Biotechnology Information Gene Expression Omnibus database, the researchers identified 233 genes whose expression in liver differed before and after liver injury (the hepatic injury and regeneration, or HIR, signature). Statistical analyses indicate that the expression of the HIR signature in archived tissue samples was significantly associated with late recurrence of HCC in three independent groups of patients, but not with early recurrence (a significant association between two variables is one that is unlikely to have arisen by chance). By contrast, a tumor-derived 65-gene signature previously developed by the researchers was significantly associated with early recurrence but not with late recurrence. Notably, as few as four genes from the HIR signature were sufficient to construct a reliable predictor for late recurrence of HCC. Finally, the researchers report that many of the genes in the HIR signature encode proteins involved in inflammation and cell death, but that others encode proteins involved in cellular growth and proliferation such as STAT3, a protein with a well-known role in liver regeneration.

**What Do These Findings Mean?:**

These findings identify a gene expression signature that was significantly associated with late recurrence of HCC in three independent groups of patients. Because most of these patients were infected with HBV, the ability of the HIR signature to predict late occurrence of HCC may be limited to HBV-related HCC and may not be generalizable to HCC related to other causes. Moreover, the predictive ability of the HIR signature needs to be tested in a prospective study in which samples are taken and analyzed at baseline and patients are followed to see whether their HCC recurs; the current retrospective study analyzed stored tissue samples. Importantly, however, the HIR signature associated with late recurrence and the 65-gene signature associated with early recurrence provide new insights into the biological differences between late and early recurrence of HCC at the molecular level. Knowing about these differences may lead to new treatments for HCC and may help clinicians choose the most appropriate treatments for their patients.

**Additional Information:**

Please access these websites via the online version of this summary at http://dx.doi.org/10.1371/journal.pmed.1001770.

- The US National Cancer Institute provides information about all aspects of cancer, including detailed information for patients and professionals about primary liver cancer (in English and Spanish)
- The American Cancer Society also provides information about liver cancer (including information on support programs and services; available in several languages)
- The UK National Health Service Choices website provides information about primary liver cancer (including a video about coping with cancer)
- Cancer Research UK (a not-for-profit organization) also provides detailed information about primary liver cancer (including information about living with primary liver cancer)
- MD Anderson Cancer Center provides information about symptoms, diagnosis, treatment, and prevention of primary liver cancer
- MedlinePlus provides links to further resources about liver cancer (in English and Spanish)

## Introduction

Liver cancer is the second-leading cause of cancer mortality worldwide, accounting for approximately 600,000 cancer-related deaths annually [1]. The incidence rate of hepatocellular carcinoma (HCC), the most common type of liver cancer, has increased in the United States recently and is expected to double in the next 10 to 20 y [2]–[4]. Despite recent advances in diagnosis and management, the median survival of patients with HCC is less than 8 mo, and the disease is still one of the most fatal cancers [4]. Surgical resection, liver transplantation, and local ablation remain the only curative modalities of HCC [5],[6], and recurrence occurs in up to 70% of patients within 5 y after resection [7],[8]. Therefore, among patients who have received curative treatment, survival is jeopardized by tumor recurrence.

In HCC, two distinct types of recurrence are known. Early recurrence arises from primary cancer cells disseminating to the surrounding liver and is usually observed within the first 2 y after surgery. In contrast, late recurrence, which is typically observed >2 y after surgery, appears to be a result of chronic liver damage known as the “field effect,” and produces de novo tumors that are independent of resected primary tumors [7]. The two types of recurrence have different clinical courses and probably appear in distinct biological contexts [9]. For better disease management, it is therefore important to uncover the biological characteristics of each type of recurrence and to develop distinct molecular prognostication systems that can identify patients at high risk for either type. Despite the importance of managing recurrence, our knowledge of the genetic alterations associated with either type, especially late recurrence, is fragmentary.

Chronic injury and inflammation are known to promote tumor development. HCC is one of the best known examples, as more than 90% of HCCs arise in the context of hepatic injury and inflammation [10]–[12]. Chronically unresolved inflammation is frequently associated with persistent hepatic injury and concurrent regeneration, which prime the liver for development of HCC [13]. This process is highly similar to a persistent wound-healing response, regardless of the differences among various etiological factors such as viruses, alcohol, and fatty liver [14],[15]. Therefore, we hypothesized that gene expression patterns significantly associated with hepatic injury and regeneration (HIR) would reflect the potential risk of HCC development as well as the de novo recurrence of HCC, and that these patterns could serve as predictive markers to identify patients with high risk of de novo recurrence after treatment.

We and other researchers have discovered several genomic predictors for recurrence of HCC [16]–[23]. In particular, one study identified a prognostic gene expression signature (the Broad signature) that was significantly associated with late recurrence of HCC in a patient population that was mostly hepatitis C virus (HCV)–positive [20]. However, it is currently unknown whether this signature is applicable to HCC associated with other etiological factors. Furthermore, molecular or genomic predictors that can predict and discriminate early and late recurrence have not been firmly established. In the current study, we aimed to analyze gene expression data from human livers undergoing liver injury with regeneration in order to develop a genomic predictor for late recurrence of HCC that is associated with hepatitis B virus (HBV) and a new prognostication model for prediction and discrimination of early and late recurrence of HCC after surgery.

## Methods

### Study Design: Patients and Cohorts

This is a retrospective multi-center cohort study aimed at investigating the association of a gene expression signature reflecting HIR in human liver with recurrence of HCC and developing a genomic predictor that can identify patients with a high risk of recurrence after surgical treatment. Archived tissue samples (tumors and matched surrounding non-tumor tissues) of 72 HCC patients (cohort 1) undergoing hepatectomy as primary treatment at the Dongsan Medical Center of Keimyung University, Daegu, Korea, and the Guro Hospital of Korea University College of Medicine, Seoul, Korea, between 26 December 2001 and 3 June 2011 were included in this study. Patients were consecutively enrolled and selected on the basis of the availability of both tumor tissues and non-tumor surrounding tissues. All liver tissues were frozen in liquid nitrogen and stored at −80°C until RNA extraction. The study protocols were approved by the Institutional Review Boards at Dongsan Medical Center, Guro Hospital, and the University of Texas MD Anderson Cancer Center, and all participants provided written informed consent. Patients in cohort 1 were followed up prospectively at least once every 3 mo after surgery. Recurrence-free survival, which was defined as the time from surgery to the first confirmed recurrence, was censored when a patient died or was alive without recurrence at last contact. To test the robustness of the genomic predictor, gene expression data from two independent cohorts were used. Gene expression and clinical data from Queen Mary Hospital of University of Hong Kong (cohort 2, *n = *96) and Fudan University in China (cohort 3, *n = *228) were obtained from Gene Expression Omnibus (GSE22058 and GSE14520, respectively) [24],[25]. Patients in the two validation cohorts underwent surgery between 1990 and 2007. The baseline characteristics of all three cohorts are shown in Table 1. The patients in the three cohorts are largely representative of those with HBV.

**Table 1:** Baseline characteristics of HCC patients.

Variable	Cohort 1 (n = 72)	Cohort 2 (n = 96)	Cohort 3 (n = 228)
**Male sex**	58 (80.6%)	78 (81.3%)	201 (88.2%)
**Median (range) age (years)**	57.5 (29–77)	55 (27–80)	50 (21–77)
**AFP >300 ng/ml**	18 (25.0%)	36 (37.5%)	104 (45.6%)
**HBV**	60 (83.3%)	84 (87.5%)	209 (91.7%)
**Liver cirrhosis**	36 (50.0%)	60 (62.5%)	211 (92.5%)
**Tumor size ≤5 cm**	45 (62.5%)	36 (37.5%)	145 (63.6%)
**Single tumor**	60 (83.3%)	71 (74.0%)	183 (80.3%)
**Vascular invasion**	24 (33.3%)	47 (49.0%)	88 (38.6%)
**BCLC stage** *			
0	5 (6.9%)	4 (4.2%)	20 (8.8%)
A	53 (73.6%)	64 (66.7%)	144 (63.2%)
B	8 (11.1%)	19 (19.8%)	22 (9.6%)
C	6 (8.3%)	9 (9.4%)	26 (11.4%)
**Median follow-up (months)**	33.1	45.7	51.9

*BCLC stage was not available for 16 patients of cohort 3.

AFP, alpha-fetoprotein.

### Gene Expression Data from Human Tissues

To identify a gene expression signature that faithfully reflects HIR in human liver, i.e., a HIR signature, we acquired gene expression data from liver biopsies of liver transplantation and hepatectomy patients from the National Center for Biotechnology Information's Gene Expression Omnibus database. The first and second sets of biopsies were taken from the livers of deceased donors (*n = *13) and living donors (*n = *8), respectively (GSE12720) [26]. The first core biopsies were taken before manipulation of the liver, and the second biopsies were taken after reperfusion following completion of bile duct anastomosis. A third set of biopsies was taken before and after surgery from remnant liver of patients who had undergone partial hepatectomy as treatment for colon cancer metastasis or hepatoblastoma (*n = *4, GSE15239).

Gene expression data from cohort 1 were generated using the Illumina microarray platform HumanHT-12 version 4. Briefly, total RNA was extracted from fresh-frozen tissues using a mirVana RNA isolation and labeling kit (Ambion). For each sample, 500 ng of total RNA was used for labeling and hybridization according to the manufacturer's protocols. After the bead chips were scanned with an Illumina BeadArray Reader, the microarray data were normalized using the quantile normalization method in the Linear Models for Microarray Data package in the R language environment [27]. The expression level of each gene was transformed into log2 base before further analysis. Primary microarray data from human liver tissues are available in the National Center for Biotechnology Information's Gene Expression Omnibus public database (accession number GSE39791).

### Selection of Genes in the Hepatic Injury and Regeneration Signature

We identified genes that were differentially expressed between two series of biopsies in three different datasets (partial hepatectomy, deceased-donor liver transplantation, and living-donor liver transplantation). Genes were considered statistically significant if their *p-*value was less than 0.005. This stringent significance threshold was used to limit the number of false-positive findings. We also performed a global test of whether the expression profiles differed between two classes (two series of biopsies) by permuting the labels of which arrays corresponded to which classes. For each permutation, the *p-*values were recomputed, and the number of genes significant at the 0.005 level was noted. The proportion of the permutations that gave at least as many significant genes as the actual data was the significance level of the global test. A total of 325 gene features (representing 233 unique genes) were identified as the HIR signature.

### Statistical Analysis

We used BRB ArrayTools for analysis of gene expression data [28]. Genes differentially expressed between the two classes of HCC were identified using a random-variance *t*-test [29]. Expression was considered statistically significant if the *p-*value was less than 0.005. To stratify HCC patients as being at low risk or high risk of de novo late recurrence of disease according to the HIR signature, we applied a classification algorithm based on a Bayesian compound covariate predictor (see Text S1) [30].

For patient stratification in our cohorts according to the 186-gene Broad surrounding tissue signature, we applied a weighted gene approach to stratify HCC patients as described in a previous study [20]. Out of the 186 genes, 170 were present in the microarray platforms from three cohorts. Gene expression data were centralized independently across all samples before they were integrated. The patients were stratified into two risk groups using a Cox score derived in a previous study [20]. Briefly, each gene was weighted using the corresponding Cox score, and all patients were ranked by the summation of gene weights. In the Broad risk score, a higher score is associated with poorer prognosis. The patients whose gene weights were higher than the lowest gene weight of the poor prognosis group in the Broad cohort were classified as the poor prognosis group; those patients whose gene weights were lower than the highest gene weights of the good prognosis group in the Broad cohort were classified in the good prognosis group.

Prediction analysis of microarrays (PAM) was carried out as described previously [31]. In addition to PAM, multivariate logistic regression analysis was carried out to find minimum genes in the prediction model. Briefly, univariate analysis was first applied to genes in the HIR signature to identify genes whose expression was significantly associated with recurrence-free survival in the pooled patient cohort (*p*<0.005), yielding 13 genes. Next, backward stepwise multivariable regression analysis was carried out to find genes as independent predictors of recurrence (*p*<0.1). Four genes (*RALGDS*, *IER3*, *CEBPD*, and *SLC2A3*) were identified from the analysis.

Patient prognoses were estimated using Kaplan–Meier plots and the log-rank test. We used multivariate Cox proportional hazards regression analysis to evaluate independent prognostic factors associated with recurrence-free survival. Recurrence-free survival was defined as the time from surgery to the first confirmed recurrence; data were censored when a patient died or was alive without recurrence at last contact. As covariates we used sex, age, alpha-fetoprotein concentration, infection with HBV, liver cirrhosis, tumor size, tumor number, vascular invasion, Barcelona Clinic Liver Cancer (BCLC) tumor stage, 65-gene risk score, and the HIR signature [32]. All statistical tests were two-tailed. A *p*<0.05 indicated statistical significance. All statistical analyses were conducted in the R language environment (http://www.r-project.org).

### Gene Network Analysis for Identification of Dominant Transcription Factors

We used the gene network analysis built into Ingenuity Pathway Analysis to identify potential upstream transcription factors that regulate gene expression patterns enriched in the HIR subtype. The analysis is based on prior knowledge of expected effects of transcriptional factors on their target genes stored in the Ingenuity Knowledge Base [33]. Briefly, the analysis examines the known targets of each transcription factor in the HIR signature and compares their direction of change (i.e., expression in the HIR subtype relative to the quiescent [QT] subtype) to what is expected from the literature. If the direction of change is consistent with the literature across the majority of targets, then the transcriptional factor is predicted to be active in the HIR subtype, whereas if the direction of change is mostly inconsistent (anti-correlated) with the literature, then the transcriptional factor is predicted to be inactive in the HIR subtype. If there is no clear pattern, then there is no prediction either way. Regulation *z*-score was used to estimate the activation state of the transcription factors. An absolute *z*-score of >2 was considered significant as suggested by Ingenuity Pathway Analysis. The overlap *p-*values generated by Fisher's exact test were used to estimate the statistical significance of overlap between the dataset genes and the genes regulated by a transcription factor.

### Immunohistochemistry

Surrounding liver tissues from 20 patients with HCC (ten with early recurrence [≤2 y] and ten with late recurrence [>2 y]) were investigated for immunohistostaining validation. For comparison, normal liver tissues obtained from nine donors for liver transplantation were used as normal control. Representative sections of formalin-fixed, paraffin-embedded tissues were used for immunohistochemistry. Primary antibodies against STAT3 (Clone 124H6, Cell Signaling Technology; dilution 1∶2,000) and p-STAT3 (Tyr705; Clone D3A7, Cell Signaling Technology; dilution 1∶200) were used. Briefly, 4-µm-thick sections of the tissues were deparaffinized and rehydrated. After treatment with a 3% hydrogen peroxide solution for 10 min to block endogenous peroxidases, the sections were pretreated in 10 mM citrate buffer (pH 6.0) in a microwave oven for 15 min for antigen retrieval. After incubation with the primary antibodies, the sections were processed using the EnVision detection system (Dako) according to the manufacturer's instructions, and 3,3′-diaminobenzidine tetrahydrochloride was used as a chromogen. All sections were counterstained with Mayer hematoxylin. For interpretation of the immunohistochemical stain results, the staining intensities of STAT3 were graded on a scale of 0 to 2 (0, negative; 1, moderately positive; 2, strongly positive), and the extent of distribution was rated on a scale of 0 to 3 (0, positive in <1%; 1, 1%–30%; 2, 31%–70%; 3, 71%–100%). For p-STAT3, the staining intensities were graded on a scale of 0 to 3 (0, negative; 1, weakly positive; 2, moderately positive; 3, strongly positive), and the extent of distribution was also rated on a scale of 0 to 3 (0, not positive; 1, 0.1%–1%; 2, 1%–5%; 3, >6%). The histoscore was determined by multiplying the intensity scores by the distribution scores (histoscore for STAT3: score 0, 0; score 1, 1–3; score 2, 4–6; histoscore for p-STAT3: score 0, 0–1; score 1, 2–4; score 2, 6–9).

### Quantitative Reverse Transcription PCR Experiments

The mRNA expression levels of 19 genes (*BIRC3, GADD45B, IL1RN, LDLR, MCL1, RALGDS, CDKN1A, CCL20, ADM, MYC, DUSP5, BCL3, SOD2, SERPINE1, PHLDA1, C13ORF15, IER3, CEBPD*, and *SLC2A3*) were quantified using quantitative reverse transcription polymerase chain reaction (qRT-PCR) experiments. Total RNAs from the surrounding non-tumor liver tissues of 24 patients in cohort 1 were reverse-transcribed using a first-strand cDNA synthesis kit (Promega, according to the manufacturer's specifications). The resulting cDNAs were assayed using OriGene primers specific to each gene and the Mastercycler ep realplex system (Eppendorf). Cycling conditions were 95°C for 30 s, followed by 40 cycles of 95°C for 5 s, 62°C for 30 s, and 72°C for 20 s. Relative amounts of mRNAs were calculated from the threshold cycle number using expression levels of GAPDH as an endogenous control. All experiments were performed in triplicate, and average values are presented.

## Results

### The Hepatic Injury and Regeneration Signature and Its Association with Recurrence of HCC

**Figure 1:**
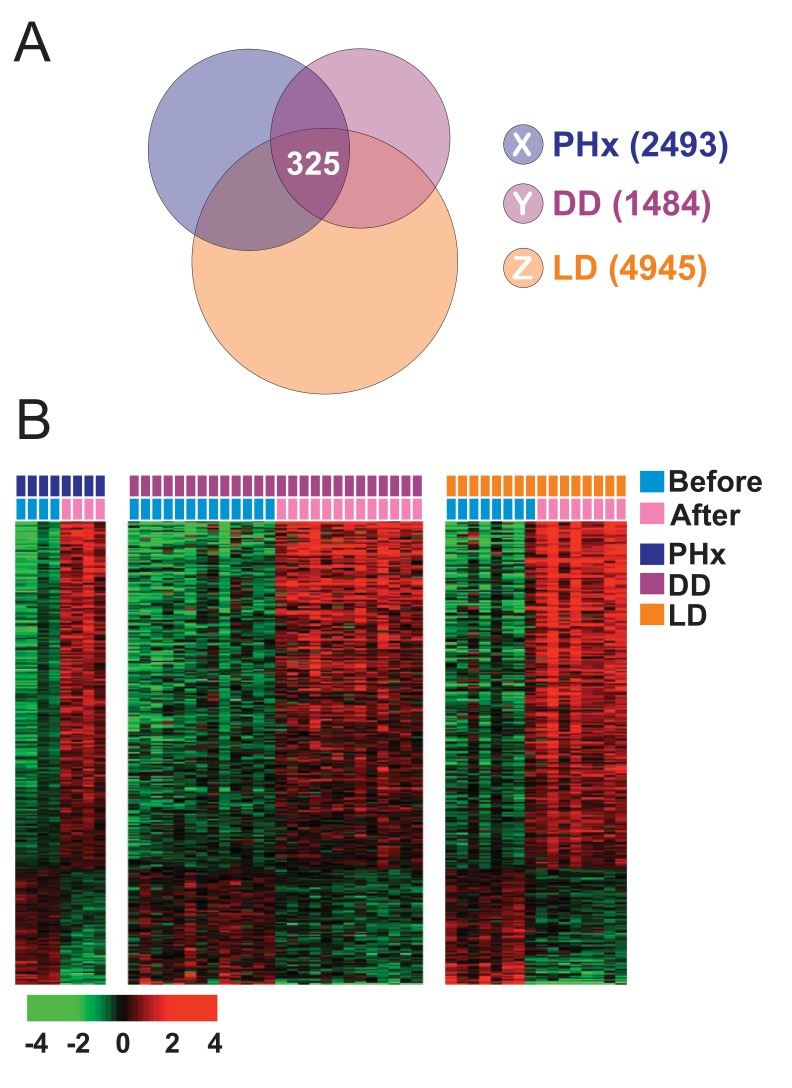
Hepatic injury and regeneration gene expression signature from human liver. (A) Venn diagram of human genes whose expression levels are significantly different before and after liver transplantation or partial hepatectomy. Three gene lists (X, Y, and Z) represent differentially expressed genes from three datasets (partial hepatectomy [PHx], deceased-donor transplantation [DD], and living-donor transplantation [LD]). A *p-*value of <0.005 was required for a gene to be retained. (B) Expression patterns of the 325 probes representing 233 unique genes shared by the three patient groups. The data are presented in matrix format, in which rows represent individual genes, and columns represent each tissue sample. Each cell in the matrix represents the expression level of a gene feature in an individual tissue sample. The colors red and green in cells reflect relatively high and low expression levels, respectively, as indicated in the scale bar (log2 transformed scale). Colored bars at the top of the heat map represent samples as indicated.

Because injury-mediated wound healing triggers hepatic regeneration and inflammation that can prime the liver for development of HCC [13], we tried to find genes whose expression patterns are highly associated with injury and regeneration in human liver. To do this, we used gene expression data from three types of injured livers: livers from patients with partial hepatectomy, livers from deceased-donor liver transplantations, and livers from living-donor liver transplantations. We first independently selected genes whose expression patterns differed significantly (*p*<0.005) before and after hepatic injury with regeneration in the three data-sets. The expression of 325 probes representing 233 unique genes (the HIR signature) was significantly altered in all three datasets (Figure 1; Table S1).

**Figure 2:**
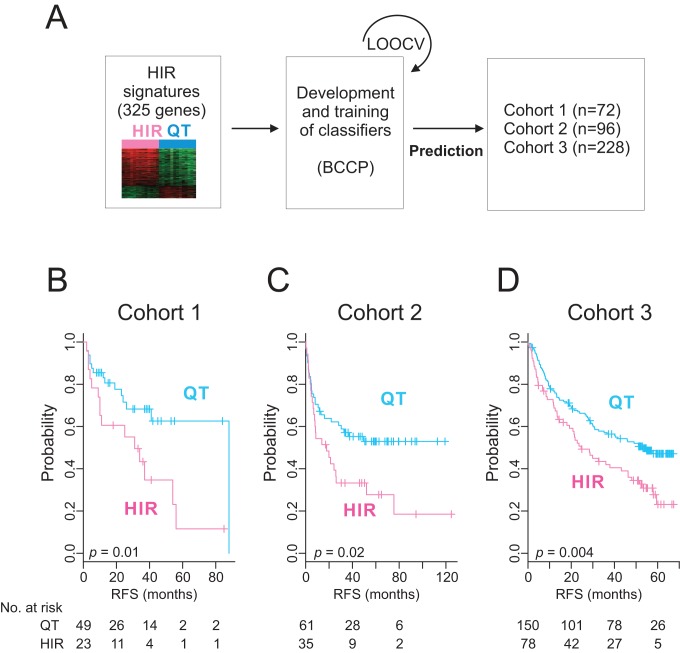
Construction of prediction models in test cohorts. (A) A schematic overview of the strategy used for constructing prediction models and evaluating predicted outcomes based on the 325-gene HIR signature. (B–D) Kaplan–Meier plots of the HIR and QT subgroups predicted by the Bayesian compound covariate predictor in cohorts 1 (B), 2 (C), and 3 (D). *p-*Values were obtained by log-rank test. The vertical lines indicate censored data. BCCP, Bayesian compound covariate predictor; LOOCV, leave-one-out cross-validation; RFS, recurrence-free survival.

Because the expression patterns of selected genes should reflect persistent HIR, mimicking the priming condition for HCC development, we hypothesized that the HIR signature would be highly associated with the development of HCC or de novo recurrence of HCC. Thus, we applied the HIR classification algorithm to gene expression data from patients' surrounding non-tumor liver tissues to test whether it could predict the recurrence of HCC after surgery. Gene expression data of surrounding tissues from the 72 patients in cohort 1 were used for this analysis, and the Bayesian compound covariate predictor algorithm [30] was applied (Figure 2A) as described in Text S1.

When patients in cohort 1 were dichotomized according to the HIR signature, 23 patients (32%) were predicted to have a high probability (>0.5) of having the HIR signature (HIR subgroup), and 49 patients (68%), a high probability of having the QT signature (QT subgroup). Recurrence-free survival differed significantly by subgroup (*p* = 0.01 by log-rank test; Figure 2B), strongly indicating that the HIR signature can identify patients with a high risk of overall recurrence after surgery.

We further tested the association of the HIR signature with recurrence in the independent cohort 2 by applying the HIR classification algorithm to gene expression data from surrounding tissues and found that recurrence-free survival between the predicted subgroups differed significantly (*p* = 0.02; Figure 2C). The robustness of the signature's association with patient prognosis was further validated in another, larger independent cohort (*p* = 0.004 in cohort 3; Figure 2D). The results from these three independent HCC cohorts clearly demonstrate a strong association between the HIR signature and HCC recurrence.

### Prediction of Late Recurrence of HCC by HIR Signature

**Figure 3:**
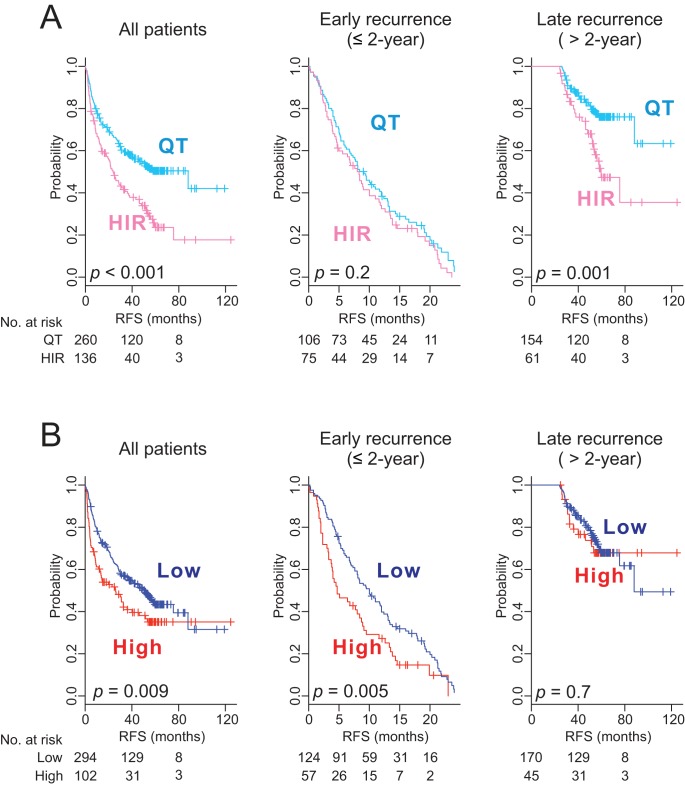
Kaplan–Meier survival plots of recurrence free survival of patients from pooled cohorts. Patients were stratified by the HIR signature (A) or the 65-gene risk score (B). All patients (*n = *396) are plotted in the left panel, patients with ≤2 y of follow-up (early recurrence) in the middle panel, and patients with more than 2 y of follow-up (late recurrence) in the right panel. *p-*Values were obtained from the log-rank test. The vertical lines denote observations that were censored owing to loss to follow-up or on the date of the last contact. RFS, recurrence-free survival.

Because de novo recurrence largely comprises late recurrence, which usually occurs >2 y after surgery [7], we carried out a subset analysis of the pooled data to assess the ability of the HIR signature to predict early and late recurrence. The signature lacked a significant association with early recurrence (recurrence ≤2 y after surgery; *p* = 0.2), but was significantly associated with late recurrence (recurrence >2 y after surgery; *p* = 0.001; Figure 3A). Furthermore, when we carried out stepwise subset analysis for recurrence more than 2 y, 3 y, and 4 y after surgery, the significance of the association of the HIR signature with late recurrence remained the same (Figure S1), providing evidence of a strong association between the HIR signature and late recurrence regardless of the cutoff time for late recurrence. This finding strengthens our hypothesis of a direct correlation of the HIR signature with de novo development of HCC.

### Prediction of Early Recurrence of HCC by a Tumor-Derived 65-Gene Risk Score

Using gene expression data from tumors, we had previously developed a 65-gene risk score that can identify patients at high risk for HCC recurrence after surgery [34]. When we applied it to gene expression data from tumors of the same patients (*n = *396), the risk score was significantly associated with a high overall recurrence rate (*p* = 0.009; Figure 3B). However, it was significantly associated with early recurrence only (*p* = 0.005) and failed to identify patients at high risk for late recurrence (*p* = 0.7), suggesting that this tumor-derived signature is more directly correlated with recurrence mediated by intrahepatic metastasis of primary tumors, which usually occurs within 2 y after surgery [7].

Because recurrence is the leading cause of death among HCC patients during the first year after surgery [35]–[37], we further divided early recurrence patients into very early (<1 y) and intermediate (between 1 and 2 y) recurrence subpopulations and assessed the ability of two different predictors, the tumor-derived 65-gene risk score and the HIR signature, to prognosticate each of the recurrence types. As we expected, the HIR signature was not significantly associated with very early (*p* = 0.7) or intermediate recurrence (*p* = 0.3) but remained significantly associated with late recurrence (*p* = 0.001) (Figure S2). The tumor-derived risk score was significantly associated with the very early recurrence group (*p*<0.001) but not with the intermediate recurrence group (*p* = 0.4) or the late recurrence group (*p* = 0.7) (Figure S2). The fact the both signatures failed to identify patients at high risk for recurrence between 1 and 2 y after surgical resection suggests that intermediate recurrence might be a mixture of metastatic and de novo recurrence.

### Clinical Significance of the Prognostic Genomic Signatures

We next assessed the prognostic association between the two predictors (the HIR signature and the 65-gene risk score) and known demographic and clinical risk factors. For the very early recurrence subgroup (recurrence less than 1 y after surgical resection), significant predictors of recurrence-free survival were the 65-gene risk score (*p* = 0.003) and BCLC stage (*p* = 0.001), which is the most frequently used clinical staging system and already a well-known risk factor for HCC recurrence (Table 2) [5]. When the 65-gene risk score was included in the multivariate model, it was the strongest predictor of recurrence-free survival (hazard ratio, 1.8; 95% confidence interval, 1.2–2.7; *p* = 0.008). The HIR signature was not a significant predictor of very early recurrence.

**Table 2:** Univariate and multivariate Cox regression analyses of recurrence-free survival of patients in two different recurrence groups.

Characteristics	Univariate Analysis		Multivariate Analysis with 65-Gene RS		Multivariate Analysis with HIR Signature	
	Hazard Ratio (95% CI)	*p*-Value	Hazard Ratio (95% CI)	*p*-Value	Hazard Ratio (95% CI)	*p*-Value
**Very early recurrence group (<1 y, ** ***n*** ** = 125** * **)**						
Patient sex (male or female)	1.1 (0.6–1.9)	0.84				
Age (>60 y or ≤60 y)	0.9 (0.6–1.3)	0.47				
AFP (>300 ng/ml or ≤300 ng/ml)	1.2 (0.8–1.7)	0.40				
Cirrhosis (yes or no)	1.2 (0.7–1.9)	0.48				
Tumor size (>5 cm or ≤5 cm)	1.6 (1.2–2.4)	0.01	1.2 (0.7–1.9)	0.34		
Multinodular tumors (yes or no)	1.4 (0.9–2.2)	0.13				
Microvessel invasion (yes or no)	2.0 (1.3–2.9)	<0.001	1.6 (1.0–2.5)	0.03		
BCLC stage (0/A or B/C)	1.9 (1.3–2.9)	0.001	1.3 (0.8–2.1)	0.25		
HIR signature# (HIR or QT)	1.0 (0.7–1.5)	0.97				
65-gene RS# (high or low)	1.8 (1.2–2.8)	0.003	1.8 (1.2–2.7)	0.008		
**Late recurrence group (>2 y, ** ***n*** ** = 215** * **)**						
Patient sex (male or female)	1.6 (0.7–3.4)	0.26				
Age (>60 y or ≤60 y)	1.0 (0.6–1.7)	0.97				
AFP (>300 ng/ml or ≤300 ng/ml)	0.7 (0.4–1.2)	0.78				
Cirrhosis (yes or no)	0.9 (0.5–1.6)	0.72				
Tumor size (>5 cm or ≤5 cm)	1.2 (0.7–2.1)	0.45				
Multinodular tumors (yes or no)	1.1 (0.6–2.3)	0.73				
Microvessel invasion (yes or no)	1.5 (0.9–2.5)	0.11				
BCLC stage (0/A or B/C)	1.8 (0.9–3.5)	0.08			1.7 (0.9–3.4)	0.11
HIR signature# (HIR or QT)	2.2 (1.3–3.8)	0.002			2.2 (1.3–3.7)	0.002
65-gene RS# (high or low)	1.1 (0.6–2.1)	0.73				

*Patients without BCLC stage were not included in multivariate analysis.

# Two subgroups of the prognostic signatures were used as covariates during analysis.

AFP, alpha-fetoprotein; RS, risk score.

In the late recurrence subgroup (recurrence more than 2 y after surgical resection), the HIR signature (*p* = 0.002) and BCLC stage (*p* = 0.08) were important predictors of recurrence-free survival (Table 2). The HIR signature was the stronger predictor in multivariate analysis (hazard ratio, 2.2; 95% confidence interval, 1.3–3.7; *p* = 0.002). The 65-gene risk score was not a significant predictor of late recurrence.

Because the BCLC staging system recommends curative modalities, such as surgery, for only selected patients with HCC (i.e., BCLC stage 0 and A), we then limited our analysis to patients whose tumors were BCLC stage 0 or A at the time of surgery (*n* = 290). The significant association between the HIR signature and late recurrence remained the same regardless of the cutoff time for late recurrence (Figure S3). Likewise, the association of the 65-gene risk score with early recurrence remained significant (Figure S4).

**Figure 4:**
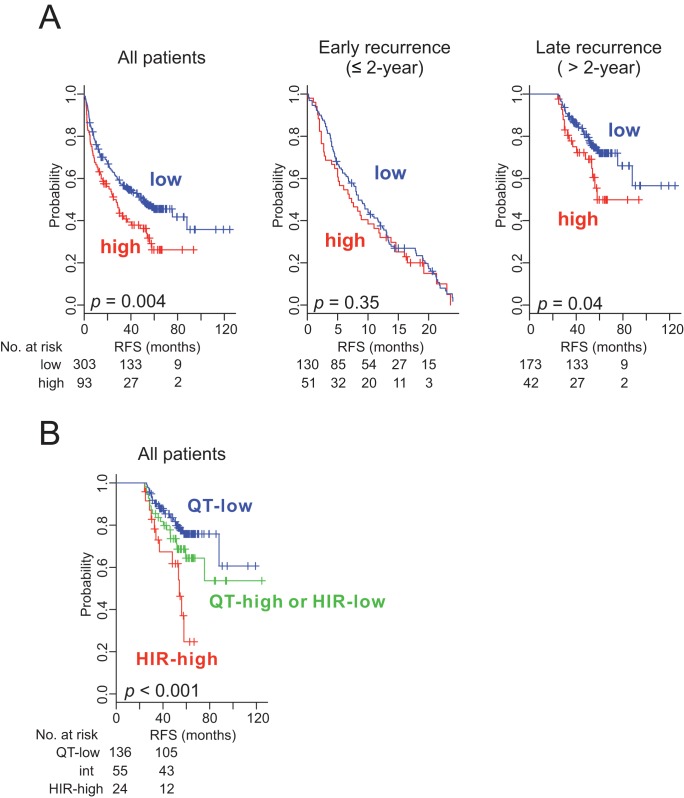
Kaplan–Meier survival plots of recurrence free survival of patients with HCC stratified by the Broad signature. (A) All patients (*n = *396) are plotted in the left panel, those with early recurrence (≤2 y) in the middle panel, and those with late recurrence (>2 y) in the right panel. (B) Patients were stratified into three groups by integrating outcomes from two prognostic models (HIR and Broad signatures); (1) HIR subgroup and Broad high-risk group (HIR-high; red), (2) QT subgroup and Broad low-risk group (QT-low; blue), and (3) QT subgroup and Broad high-risk group or HIR subgroup and Broad low-risk group (QT-high or HIR-low; int; green). *p-*Values were obtained from the log-rank test. Vertical lines denote observations that were censored owing to loss to follow-up or on the date of the last contact.

We next examined the concordance of the HIR signature with a previously developed 186-gene prognostic signature predicting late recurrence called the Broad signature [20]. When the Broad classification algorithm was applied to our cohort, 93 patients were classified into the high-risk group for late recurrence. Consistent with a previous report [20], patients in the Broad high-risk group had significantly worse recurrence-free survival rates (both overall and for late recurrence) (Figure 4A). The outcomes of the two independent prognostic models showed moderate concordance: of the 136 patients in the HIR subgroup, 54 were identified as being at high risk by the Broad signature (*r* = 0.27 by Cramer V statistics; *p*<0.001) (Table S2). It is also interesting that only four genes overlapped in both prognostic signatures, suggesting that the two signatures may capture different biological characteristics associated with late recurrence of HCC. This notion was supported by the improved prognostication when the outcomes of both prognostic models were integrated (Figure 4B). Furthermore, the hazard ratio for late recurrence in the high-risk group was substantially increased in the integrated model regardless of the cutoff for defining late recurrence (Table 3).

**Table 3:** Hazard ratios of high-risk groups in three prognostic models.

Model	Recurrence after 2 y		Recurrence after 3 y		Recurrence after 4 y	
	Hazard Ratio (95% CI)	*p*-Value	Hazard Ratio (95% CI)	*p*-Value	Hazard Ratio (95% CI)	*p*-Value
HIR signature (HIR or QT)	2.2 (1.3–3.8)	0.002	2.6 (1.3–5.4)	0.007	3.2 (1.2–8.5)	0.01
BROAD signature (high risk or low risk)	1.8 (1.0–3.2)	0.04	1.6 (0.7–3.7)	0.25	1.9 (0.6–5.9)	0.26
Combined signature (HIR and Broad high-risk or QT and Broad low-risk)	3.4 (1.7–6.6)	<0.001	4.1 (1.5–10.5)	0.004	6.0 (1.7–10.7)	0.004

### Biological Insights of Early and Late Recurrence Signatures

**Figure 5:**
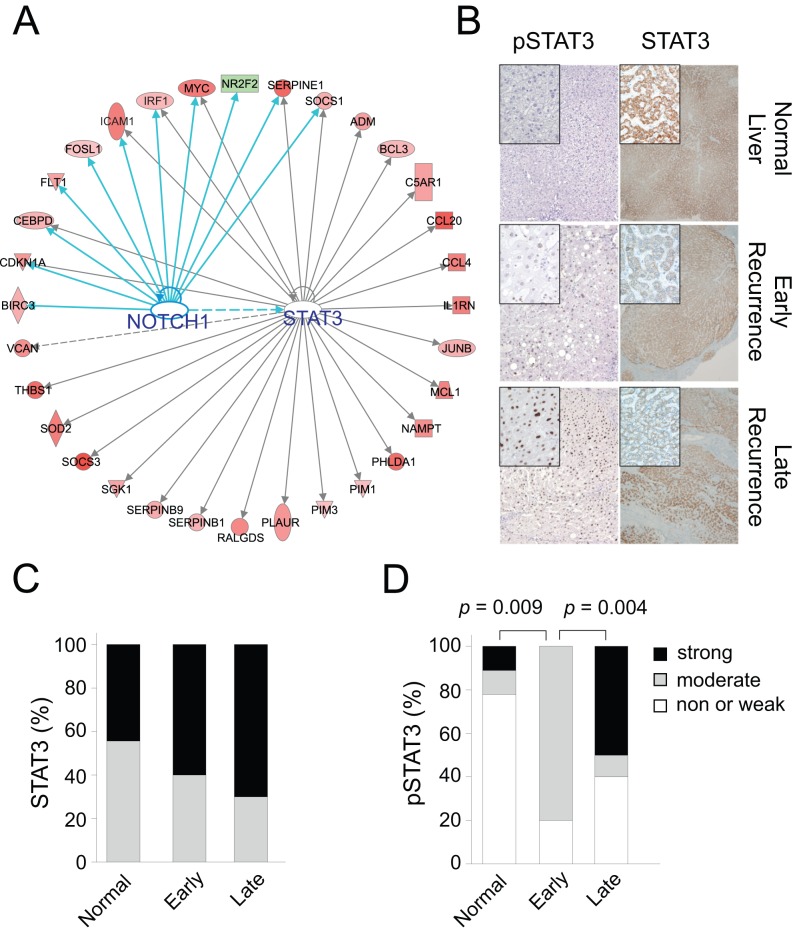
STAT3 and NOTCH1 networks in the hepatic injury and regeneration signature. (A) Ingenuity transcription factor analysis revealed that networks of genes considerably associated with STAT3 and NOTCH1 in the HIR signature. Upregulated and downregulated genes in the HIR signature are indicated by red and green, respectively. Lines and arrows represent functional and physical interactions and the direction of regulation as indicated from the literature. (B) Representative immunohistochemical staining of pSTAT3 (left panel) and STAT3 (right panel) in normal liver, surrounding non-tumor tissue of HCC with early recurrence, and surrounding non-tumor tissue of HCC with late recurrence. (C and D) Expression and phosphorylation (Y705) levels of STAT3 were compared among normal liver, surrounding non-tumor tissue of HCC with early recurrence, and surrounding non-tumor tissue of HCC with late recurrence. *p-*Values were obtained by Fisher's exact test.

To gain better insight into the molecular characteristics of the HIR signature, we categorized genes according to their known functions based on the Ingenuity Knowledge Base repository [33]. As we expected, many genes associated with the inflammatory response, inflammatory disease, and cell death were significantly enriched in the signature (Table S3), suggesting that the HIR signature reflects well the tissue damage that occurs during liver transplantation and hepatectomy. Other significantly enriched categories were cellular growth and proliferation, cancer, cell cycle, and cellular movement, which suggests that the signature also reflects the initiation of regeneration. In line with previous observations indicating a close association of HIR with the development and progression of HCC [38]–[42], many functional categories of genes in the signature are closely related to cancer development. We next carried out gene network analysis of the 233 genes in the HIR signature to uncover potential upstream regulators of the HIR signature, and discovered strong over-representation of five transcription factors, NOTCH1, STAT3, PDX1, TP53, and RELA (Table S4). Functional connectivity of the gene network further identified potential interaction between NOTCH1 and STAT3 (Figure 5A). This observation is in good agreement with the well-known role of STAT3 in liver regeneration and the potential interaction between the STAT3 and NOTCH pathways [43]–[45].

We also assessed genes whose expression in surrounding non-tumor liver tissues from patients in all three cohorts was significantly different between the HIR and QT subgroups (*p*<0.001 by two-sample *t*-test) and identified 177 genes (Figure S5). We then examined the activation status of transcription factors in the HIR subgroup. STAT3 (*p*<0.001) and NOTCH1 (*p*<0.001) were again significantly activated in the HIR subgroup (Figure S6), strongly supporting the idea that these transcription factors and the signaling pathways associated with them play key roles in late de novo recurrence of HCC. To further validate the activation of STAT3 in surrounding non-tumor tissues in HCC with late recurrence, we carried out immunohistostaining of STAT3 and p-STAT3, an indicator of STAT3 activation [46], with normal liver tissue, surrounding tissues of HCC with early recurrence (<1 y), and surrounding tissues of HCC with late recurrence (>3 y). Activation of STAT3 was significantly higher in surrounding tissues of HCC with late recurrence than in surrounding tissues of HCC with early recurrence or in normal liver tissue (Figure 5B–5D; Table S5). In addition, we also remeasured expression of downstream targets of *NOTCH1* (*MYC*, *BIRC3*, *CDKN1A*, *CEBPD*, and *SERPINE1*) in surrounding tissues using qRT-PCR methods. Consistent with our prediction, expression of downstream targets was significantly higher in HIR subtype than QT subtype samples (Figure S7), suggesting that transcriptional activity of NOTCH1 might be more active in HIR subtype. Taken together, these data strongly support our prediction based on gene network analysis.

We next carried out similar analysis with the 65 genes of the risk score associated with early recurrence. Unlike the HIR signature, the vast majority of the genes in the risk score were in functional categories related to angiogenesis and invasion (Table S6), supporting the notion that early recurrence is largely mediated by intrahepatic metastasis. Taken together, these results strongly suggest that the 65-gene risk score and the HIR signature reflect well the biological characteristics or mechanisms that are accountable for two distinct types of HCC recurrence, and that they can be used to identify patients at risk for early or late recurrence, respectively.

### Minimum Number of Genes for a Prognostic Model

**Figure 6:**
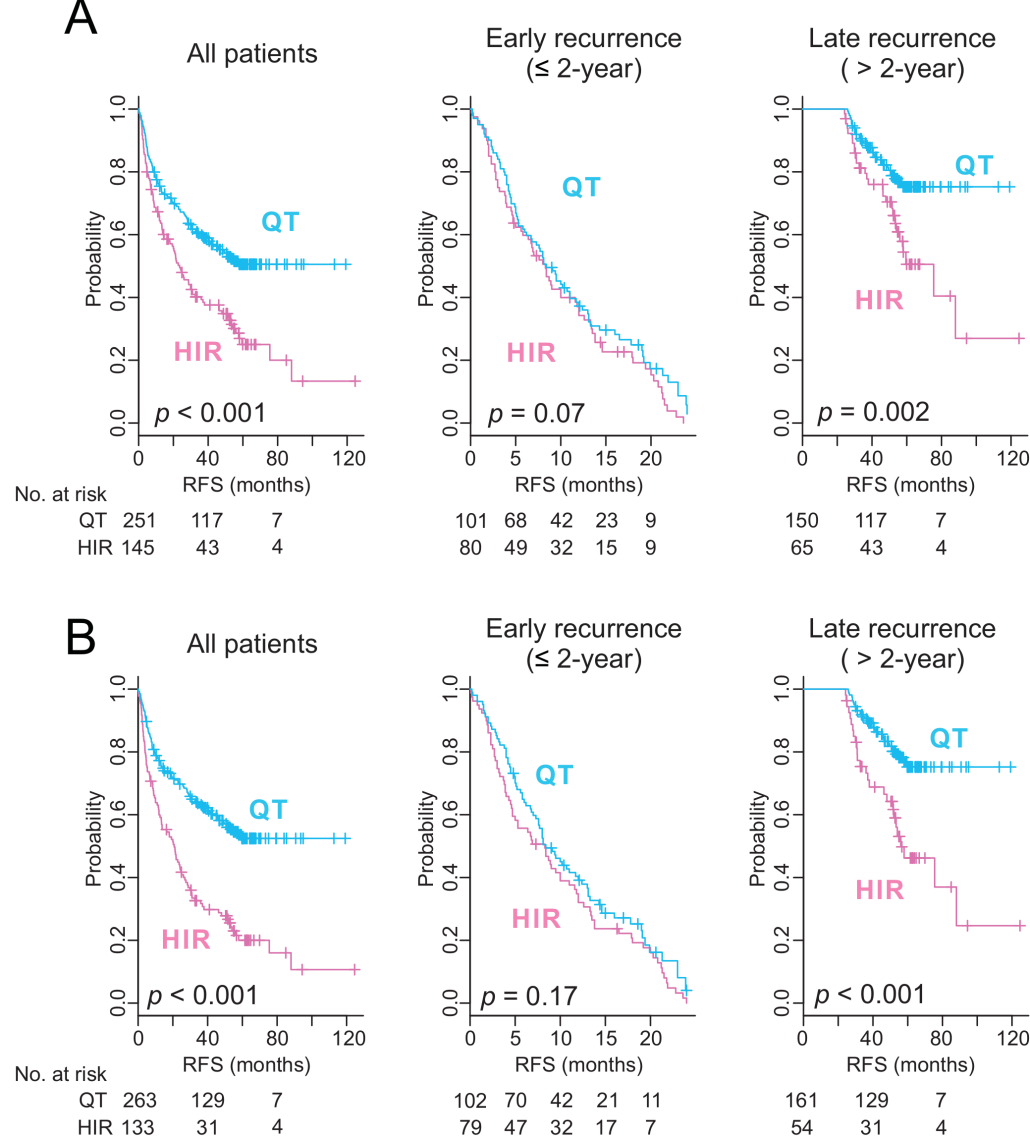
Concordance of HIR20 and HIR4 models with original HIR model. Patients were stratified by HIR20 model (A) or HIR4 model (B). All patients (*n = *396) are plotted in the left panel, those with early recurrence (≤2 y) in the middle panel, and those with late recurrence (>2 y) in the right panel. *p-*Values were obtained from the log-rank test. Vertical lines denote observations that were censored owing to loss to follow-up or on the date of the last contact.

Because the number of genes in the HIR signature (233 genes) is too large for easy translation of current findings into clinical practice, we estimated the minimum number of genes that can faithfully predict the HIR and QT subgroups. When the PAM algorithm that can find subsets of genes that best characterize each class [31] was applied to the HIR signature, ten to 20 genes were estimated to be sufficient to construct reliable prediction models with a 10% miscalculation rate (Figure S8). To confirm this prediction, we randomly selected 20 genes from the top ten functional categories in Table S3 (two genes per category) (Table S7) and constructed a prediction model with these 20 genes (HIR20 model). Consistent with PAM, the miscalculation rate of the HIR20 model for the HIR subgroup was 8%, and the concordance of the HIR20 model with the original model was significantly high (*r* = 0.84 by Cramer V statistics, *p*<0.001) (Table S8). Furthermore, the prognostic significance of the HIR20 model was very similar to that of the original model (Figure 6A).

As an independent approach to finding the minimum number of genes needed in the prediction model, we next carried out multivariate logistic regression analysis. It identified four genes (*RALGDS*, *IER3*, *CEBPD*, and *SLC2A3*) as independent predictors of recurrence. We next tested whether the expression data of these four genes would be sufficient to construct a reliable prognostic model. Similar to the HIR20 model, the four-gene prognostic model (HIR4) showed significant concordance with original model (*r* = 0.64 by Cramer V statistics, *p*<0.001) (Table S9). The prognostic significance of the HIR4 model was also highly similar to those of the HIR20 and original models (Figure 6B). Taken together, our data strongly suggest that four to 20 genes is sufficient to identify patients at high risk for late recurrence.

We next assessed the reproducibility of the gene expression measurements in our microarray study by carrying out qRT-PCR experiments with RNAs from cohort 1. For 15 selected genes in the HIR signature, expression from qRT-PCR experiments was significantly correlated with the microarray results (Figure S9). In particular, the correlations of 11 genes (*BIRC3, GADD45B, IL1RN, LDLR*, *CDKN1A, CCL20, DUSP5, BCL3, SERPINE1, PHLDA1*, and *C13ORF15*) were extremely high (*r*>0.8; *p*<0.001), suggesting that the expression data of most genes would be highly reproducible. In addition, our data demonstrated high concordance of four genes selected from multivariate logistic regression analysis (Figure S10), further suggesting that a prediction model can be developed with expression data from simpler technology such as qRT-PCR.

## Discussion

By analyzing gene expression data from human liver undergoing liver injury and regeneration that mimics the wound-healing process, and surrounding non-tumor liver tissues of patients with HCC, we identified a signature that was significantly associated with late recurrence (>2 y) after resection of the primary tumor. The robustness of this signature was validated using three independent cohorts with a total of 396 patients. Our results also suggest that signaling pathways activated during HIR may play important roles in de novo late recurrence of HCC, and are potential explorable therapeutic targets for prevention of de novo recurrence of HCC.

We further demonstrated that our previously developed tumor-derived 65-gene risk score was strongest for predicting very early recurrence (<1 y). Thus, genetic information from tumors may be limited for predicting very early recurrence, but surrounding non-tumor liver tissues may contain genetic information sufficient for predicting late recurrence. These findings support a previously developed notion that early recurrence might be largely due to metastatic tumor regrowth of primary tumor while late recurrence might be due to de novo tumor development from non-cancerous lesions with predisposing risk [7],[9].

A significant association of STAT3 activation with late recurrence was suggested by the gene network analysis with the HIR signature and later validated by immunohistostaining of the surrounding liver tissues. Our findings are in good agreement with previous studies demonstrating that STAT3 is necessary for both liver regeneration following hepatic injury and hepatocarcinogenesis in mouse models [43],[45]. The importance of STAT3 in de novo recurrence is supported by the concordant high expression of interleukin 6 (IL6) [46], a key upstream regulator of STAT3 in the liver. An earlier study demonstrated that IL6 is accountable for a higher incident rate of HCC in males than in females in a mouse model [47], suggesting that higher IL6 expression and the subsequent activation of STAT3 in surrounding liver “prime” events for tumor development. STAT3 can be also activated by other cytokines such as IL22 and leptin [48],[49], suggesting that diverse pathways might be involved in activation of STAT3 and increase susceptibility to de novo recurrence. The concomitant activation of NOTCH1, and its potential cross-talk with STAT3 in the HIR subgroup, is also intriguing because of the roles of both proteins in stem cells and organ regeneration [50]. Previous studies also support potential roles of other identified genes in the prediction model. For example, higher expression of RALGDS in HCC was previously reported, and silencing or inhibition of RALGDS significantly reduced tumorigenesis in an animal model [51]. Higher expression of CCL20 and SOCS3 was significantly associated with poor prognosis after curative resection of HCC tumors [52],[53]. Our data provide evidence for the current notion postulating that micrometastasis and de novo development are accountable for early and late recurrence, respectively, and suggest an update to the notion in recognition of the distinct molecular mechanisms for each type of recurrence that are not shared.

A previous study identified a prognostic 186-gene expression signature (the Broad signature) from surrounding non-tumor liver tissues of HCC patients that was significantly associated with late recurrence of HCC [20]. Only a few genes were shared between the independently developed HIR and Broad signatures of late recurrence, and the outcomes of the two prognostic models showed only moderate concordance. Interestingly, prognostication was substantially improved when the outcomes of both prognostic models were integrated, suggesting that each signature captures different biological characteristics that may be equally important in late recurrence of HCC. However, we cannot rule out the possibility that differences in the technological platforms account for there being only moderate concordance between the two predictors: the Broad signature was developed using a DASL (DNA annealing, selection, and ligation) microarray platform and RNA from formalin-fixed paraffin-embedded tissue, whereas the HIR signature was developed using the Affymetrix microarray platform and RNA from fresh-frozen tissues. In addition, there are substantial differences in the patient populations of the two studies. Patients in the Broad study were largely HCV-positive, while the patients in our study were mostly HBV-positive. Therefore, differences in etiologies between the two patient cohorts might be another contributing factor accountable for the only moderate concordance between the two models.

We also assessed the minimum number of genes required for a reliable prediction model by several independent approaches. First, PAM showed that ten to 20 genes would be sufficient to construct a reliable predictor with an approximately 10% miscalculation rate. Second, we found that a 20-gene predictor (HIR20 model) worked as well as the full 233-gene HIR predictor in identifying patients at high risk for late recurrence. Third, multivariate logistic regression analysis revealed that as few as four genes (HIR4 model) were sufficient to construct a reliable predictor. Lastly, the robustness of microarray data was validated by qRT-PCR experiments with the same RNAs used for microarray experiments. Thus, our study also clearly demonstrated the feasibility of developing reliable predictors with a small number of genes with the use of relatively simple technology such as qRT-PCR.

Our study has some limitations. Tissues and clinical data were retrospectively collected. Thus, our models need to be validated in a prospective study, although we obtained the same results for three independent patient cohorts. It is also important to point out that prognostication of the HIR signature may be limited to HBV-related HCC because the majority of the patients in our study were HBV-positive. This could limit the generalizability of our results. While the validation of the HIR signature in three independent cohorts of patients with different ethnic and environmental backgrounds enrolled in Korea and China strongly supports the potential generalizability of our models, it will be necessary to test our models in patients with different etiological backgrounds such as HCV or obesity in a future study.

In summary, we showed that two different genomic predictors can identify patients at high risk for early and late HCC recurrence. Because these recurrences are clinically different entities with distinctive biological characteristics, separate rational management or treatment recommendations can be developed. For example, patients at high risk of late recurrence may benefit from the use of JAK/STAT and NOTCH1 pathway inhibitors after surgical resection [54],[55]. Because current staging systems and biomarkers are limited in their ability to assess patients' risk of recurrence and their potential benefit from adjuvant therapy, two genomic predictors represent tools that could help refine treatment decisions based on molecular profiles.

## Supporting Information

Checklist S1 STARD checklist.
(DOC)

Figure S1 Kaplan–Meier survival plots of recurrence-free survival of patients with late recurrence.
(PDF)

Figure S2 Kaplan–Meier survival plots of recurrence-free survival of patients in three recurrence groups.
(PDF)

Figure S3 Kaplan–Meier survival plots of recurrence-free survival of patients with early HCC recurrence stratified by the HIR signature.
(PDF)

Figure S4 Kaplan–Meier survival plots of recurrence-free survival of patients with early HCC recurrence stratified by 65-gene risk score.
(PDF)

Figure S5 Differentially expressed genes between HIR and QT subgroups of HCC in three cohorts.
(PDF)

Figure S6 STAT3 and NOTCH1 networks in HIR subgroup of surrounding non-tumor tissues from HCC patients.
(PDF)

Figure S7 Expression of genes regulated by NOTCH1 in surrounding non-tumor tissues from HCC patients.
(PDF)

Figure S8 Miscalculation rate of prediction models with a given number of genes.
(PDF)

Figure S9 Significance concordance between expression data from microarray experiments and qRT-PCR experiments in 15 genes selected for validation of microarray data.
(PDF)

Figure S10 Significance concordance between expression data from microarray experiments and qRT-PCR experiments in four genes selected from multivariate logistic regression analysis.
(PDF)

Table S1 Genes in the HIR signature.
(DOCX)

Table S2 Overlap of predicted outcomes by two genomic predictors.
(DOCX)

Table S3 Functional categories of HIR signature
(DOCX)

Table S4 Transcription factors activated in HIR gene expression signature.
(DOCX)

Table S5 Expression and phosphorylation of STAT3 in liver tissues.
(DOCX)

Table S6 Functional categories of genes in the 65-gene risk score.
(DOCX)

Table S7 Selected 20 genes from top ten functional categories.
(DOCX)

Table S8 Concordance between the HIR and HIR20 models.
(DOCX)

Table S9 Concordance between the HIR and HIR4 models.
(DOCX)

Text S1 Supporting text.
(DOCX)

## Abbreviations

BCLC: Barcelona Clinic Liver Cancer
HBV: hepatitis B virus
HCC: hepatocellular carcinoma
HCV: hepatitis C virus
HIR: hepatic injury and regeneration
PAM: prediction analysis of microarrays
qRT-PCR: quantitative reverse transcription polymerase chain reaction
QT: quiescent
