# Supplementary material for: Genomic Predictors for Recurrence Patterns of Hepatocellular Carcinoma: Model Derivation and Validation
Source: PLoS Med. 2014 Dec 23;11(12):e1001770. doi: 10.1371/journal.pmed.1001770 (PMC4275163; doi:10.1371/journal.pmed.1001770)
Supplement: Figure S1 — Kaplan–Meier survival plots of recurrence-free survival of patients with late recurrence. (PDF) [file pmed.1001770.s002.pdf]

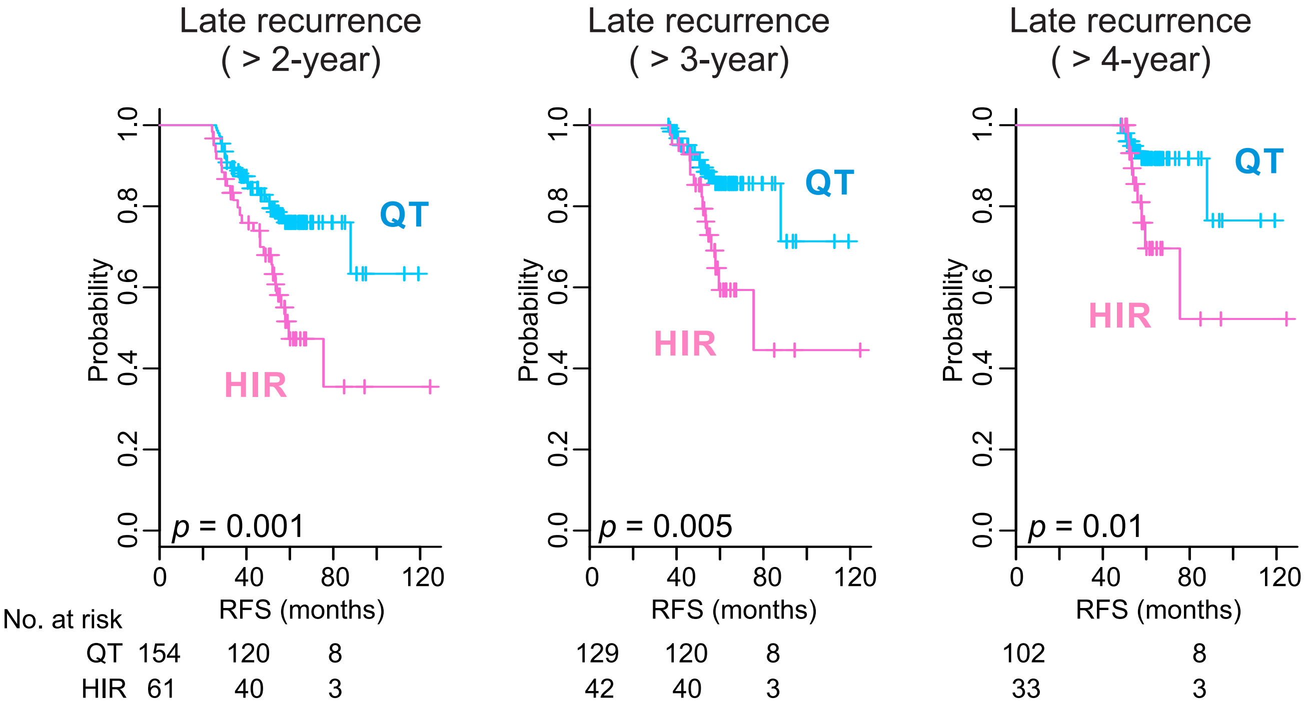

Figure S1. Kaplan–Meier Survival Plots of Recurrence Free Survival of Patients with late recurrence.

Patients with recurrence after 2 years, 3 years, and 4 years after surgery are plotted in three panels as indicated. *p* values were obtained from the log-rank test. The + symbol denotes observations that were censored owing to loss to follow-up or on the date of the last contact.
