# Supplementary material for: Genomic Predictors for Recurrence Patterns of Hepatocellular Carcinoma: Model Derivation and Validation
Source: PLoS Med. 2014 Dec 23;11(12):e1001770. doi: 10.1371/journal.pmed.1001770 (PMC4275163; doi:10.1371/journal.pmed.1001770)
Supplement: Figure S2 — Kaplan–Meier survival plots of recurrence-free survival of patients in three recurrence groups. (PDF) [file pmed.1001770.s003.pdf]

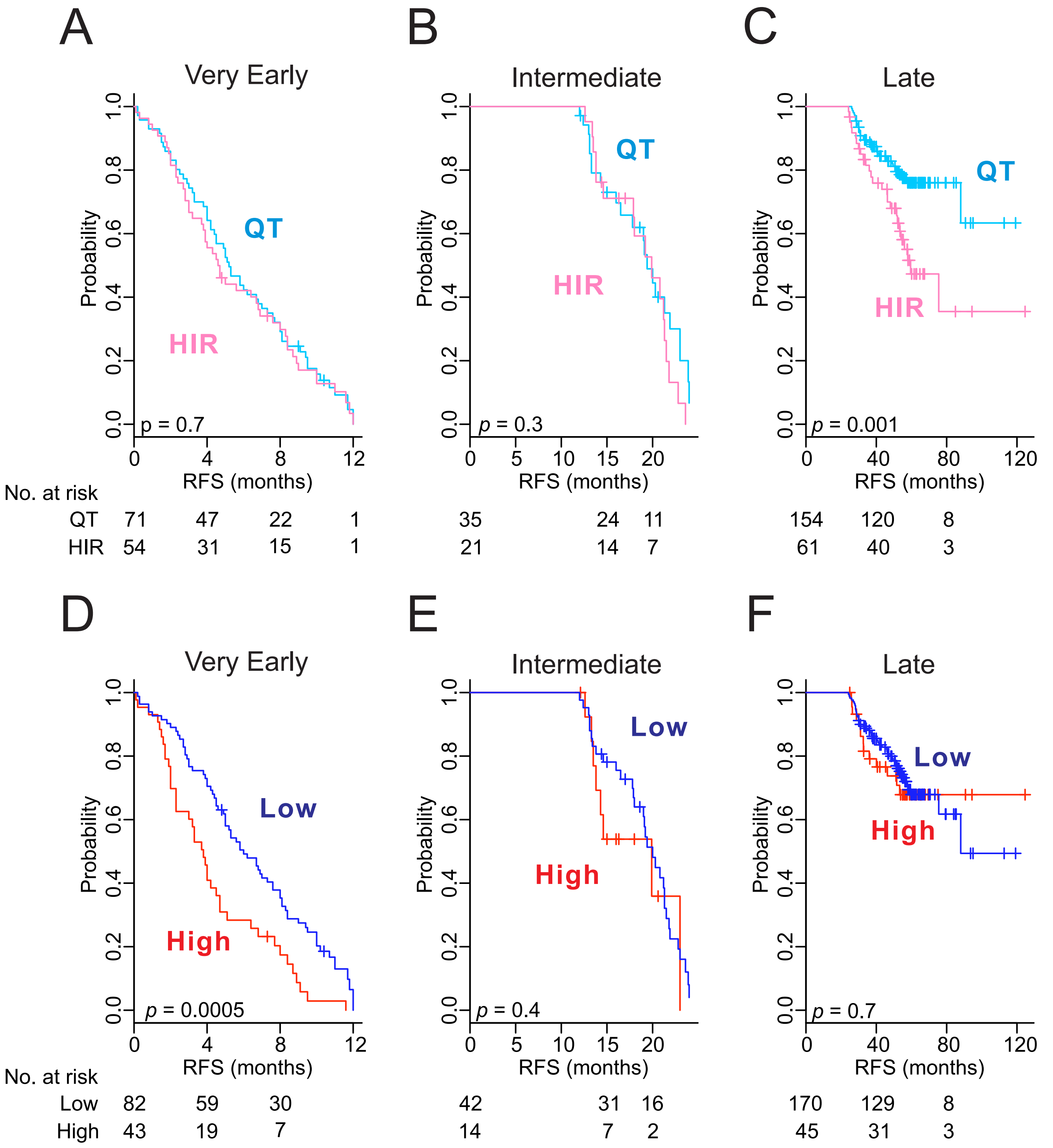

Figure S2. Kaplan–Meier Survival Plots of Recurrence Free Survival of Patients in three recurrence groups.

Patients (n = 396) were stratified by the HIR signature (A,B,C) or the 65-gene risk score (D,E,F) into very early recurrence (<1 year; A,D), intermediate recurrence (1 to 2 years; B,E), or late recurrence (>2 years; C,F). P values were obtained from the log-rank test. The + symbol denotes observations that were censored owing to loss to follow-up or on the date of the last contact. RFS, recurrence free survival; HIR, hepatic injury and regeneration subgroup; QT, quiescent subgroup.
