# Supplementary material for: Genomic Predictors for Recurrence Patterns of Hepatocellular Carcinoma: Model Derivation and Validation
Source: PLoS Med. 2014 Dec 23;11(12):e1001770. doi: 10.1371/journal.pmed.1001770 (PMC4275163; doi:10.1371/journal.pmed.1001770)
Supplement: Figure S4 — Kaplan–Meier survival plots of recurrence-free survival of patients with early HCC recurrence stratified by 65-gene risk score. (PDF) [file pmed.1001770.s005.pdf]

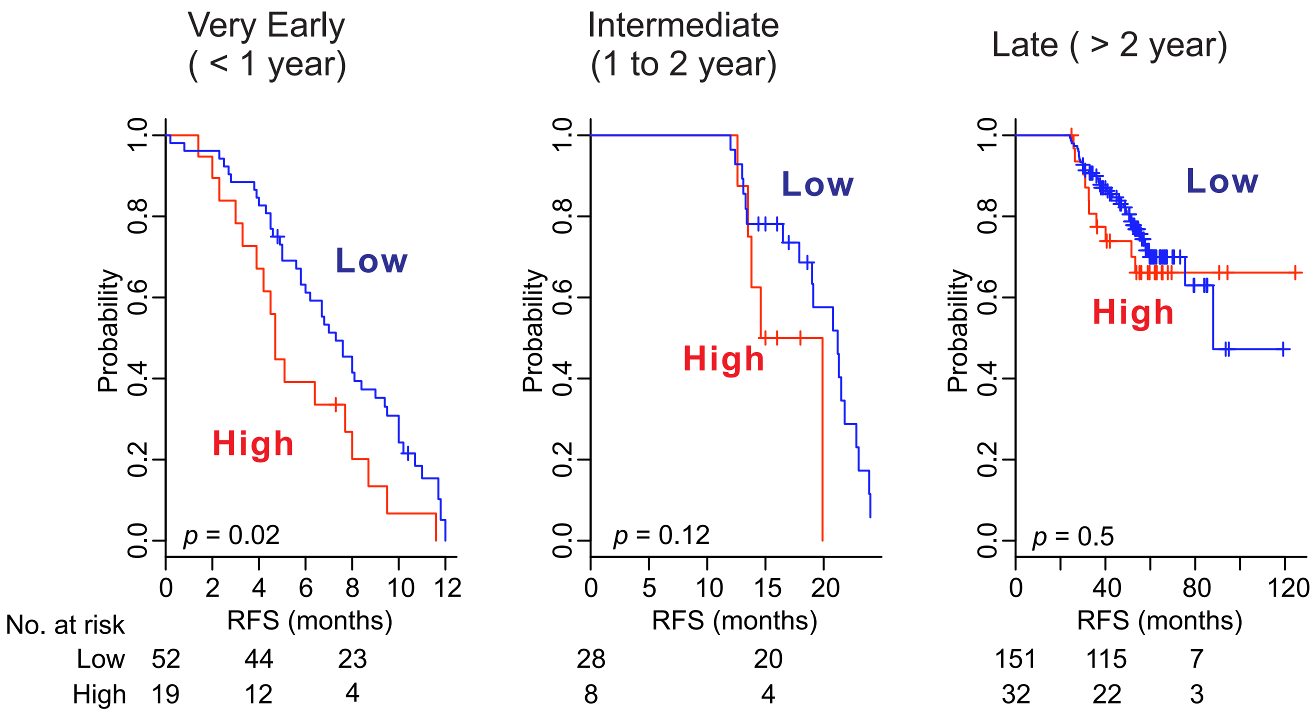

Figure S4. Kaplan–Meier Survival Plots of Recurrence Free Survival of Patients with early HCC stratified by RS65.

Patients with early stage HCC (Barcelona Clinic Liver Cancer [BCLC] stage 0 and A) (n=290) were stratified by the 65-gene risk score Patients with very early recurrence (<1 year) are plotted in left panels, those with intermediate recurrence (1 to 2 years) in middle panels, and those with late recurrence (>2 years) in right panels. *P* values were obtained from the log-rank test. The + symbol denotes observations that were censored owing to loss to follow-up or on the date of the last contact.
