# Supplementary material for: Genomic Predictors for Recurrence Patterns of Hepatocellular Carcinoma: Model Derivation and Validation
Source: PLoS Med. 2014 Dec 23;11(12):e1001770. doi: 10.1371/journal.pmed.1001770 (PMC4275163; doi:10.1371/journal.pmed.1001770)
Supplement: Figure S5 — Differentially expressed genes between HIR and QT subgroups of HCC in three cohorts. (PDF) [file pmed.1001770.s006.pdf]

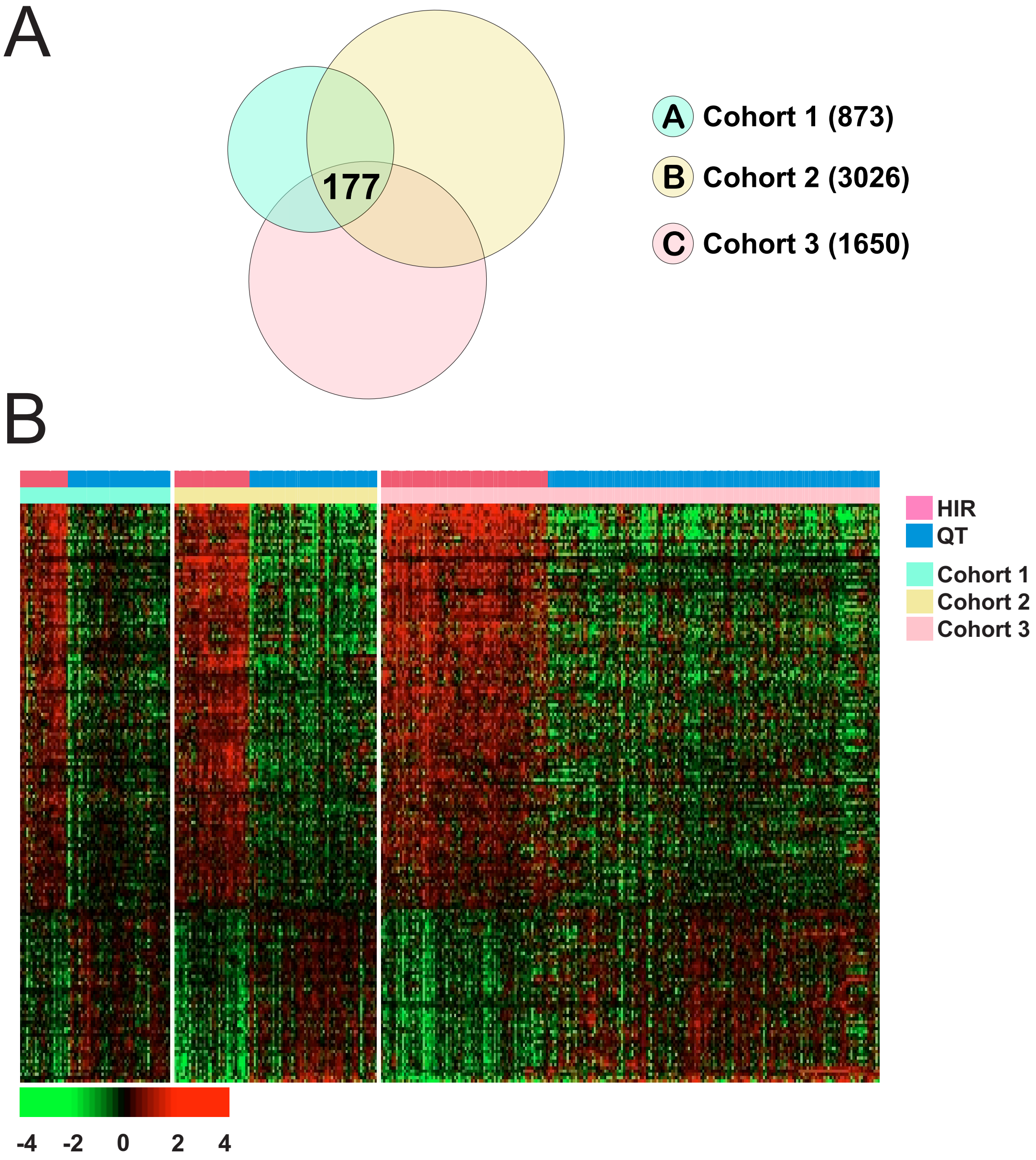

Figure S5. Differentially Expressed Genes between HIR and QT subgroups of HCC in three cohorts

(**A**) Venn Diagram of genes whose expression levels are significantly different between the HIR and QT subgroups of non-tumor surrounding tissues from patients with HCC (cohorts 1, 2, and 3). A P value of < 0.001 was required for a gene to be retained.

(**B**) Expression patterns of the 177 up- or down-regulated in all three cohorts. Colored bars at the top of the heat map represent samples as indicated.
