# Supplementary material for: Genomic Predictors for Recurrence Patterns of Hepatocellular Carcinoma: Model Derivation and Validation
Source: PLoS Med. 2014 Dec 23;11(12):e1001770. doi: 10.1371/journal.pmed.1001770 (PMC4275163; doi:10.1371/journal.pmed.1001770)
Supplement: Figure S6 — STAT3 and NOTCH1 networks in HIR subgroup of surrounding non-tumor tissues from HCC patients. (PDF) [file pmed.1001770.s007.pdf]

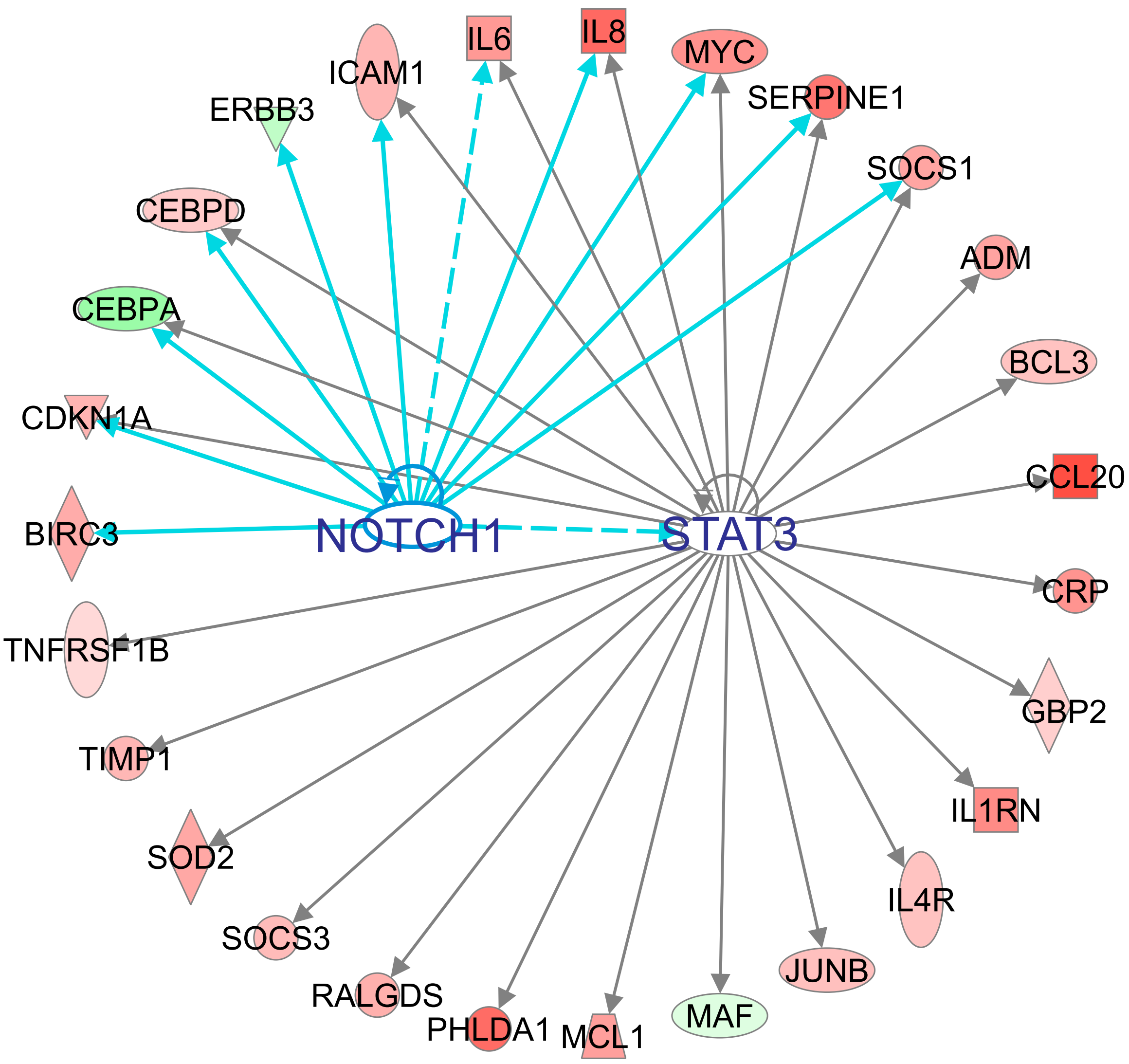

Figure S6. STAT3 and NOTCH1 Networks in HIR Subgroup of Surrounding Tissues from HCC Patients.

Networks of genes considerably associated with STAT3 and NOTCH1 in the HIR subgroup. Up-regulated and down-regulated genes are indicated by red and green, respectively. The lines and arrows represent functional and physical interactions and the directions of regulation as indicated the literature. Empty nods indicate genes not included in shared gene list.
