# Supplementary material for: Genomic Predictors for Recurrence Patterns of Hepatocellular Carcinoma: Model Derivation and Validation
Source: PLoS Med. 2014 Dec 23;11(12):e1001770. doi: 10.1371/journal.pmed.1001770 (PMC4275163; doi:10.1371/journal.pmed.1001770)
Supplement: Figure S7 — Expression of genes regulated by NOTCH1 in surrounding non-tumor tissues from HCC patients. (PDF) [file pmed.1001770.s008.pdf]

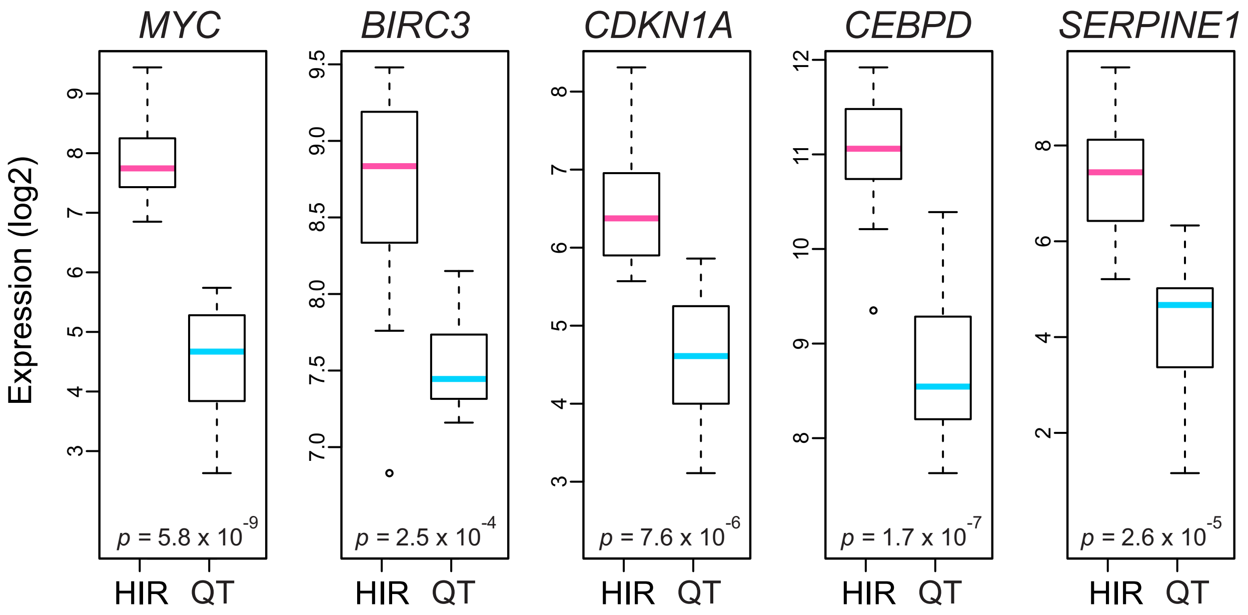

Figure S7. Expression of genes regulated by NOTCH1 in surrounding tissues from HCC patients.

Expression of 5 downstream targets of NOTCH1 was measured from 24 surrounding tissues (12 HIR and 12 QT subtypes from cohort 1) by qRT-PCR experiments. Gene expression data from qRT-PCR experiments were normalized to the expression level of *GAPDH* in the sample.
