# Supplementary material for: Genomic Predictors for Recurrence Patterns of Hepatocellular Carcinoma: Model Derivation and Validation
Source: PLoS Med. 2014 Dec 23;11(12):e1001770. doi: 10.1371/journal.pmed.1001770 (PMC4275163; doi:10.1371/journal.pmed.1001770)
Supplement: Figure S8 — Miscalculation rate of prediction models with a given number of genes. (PDF) [file pmed.1001770.s009.pdf]

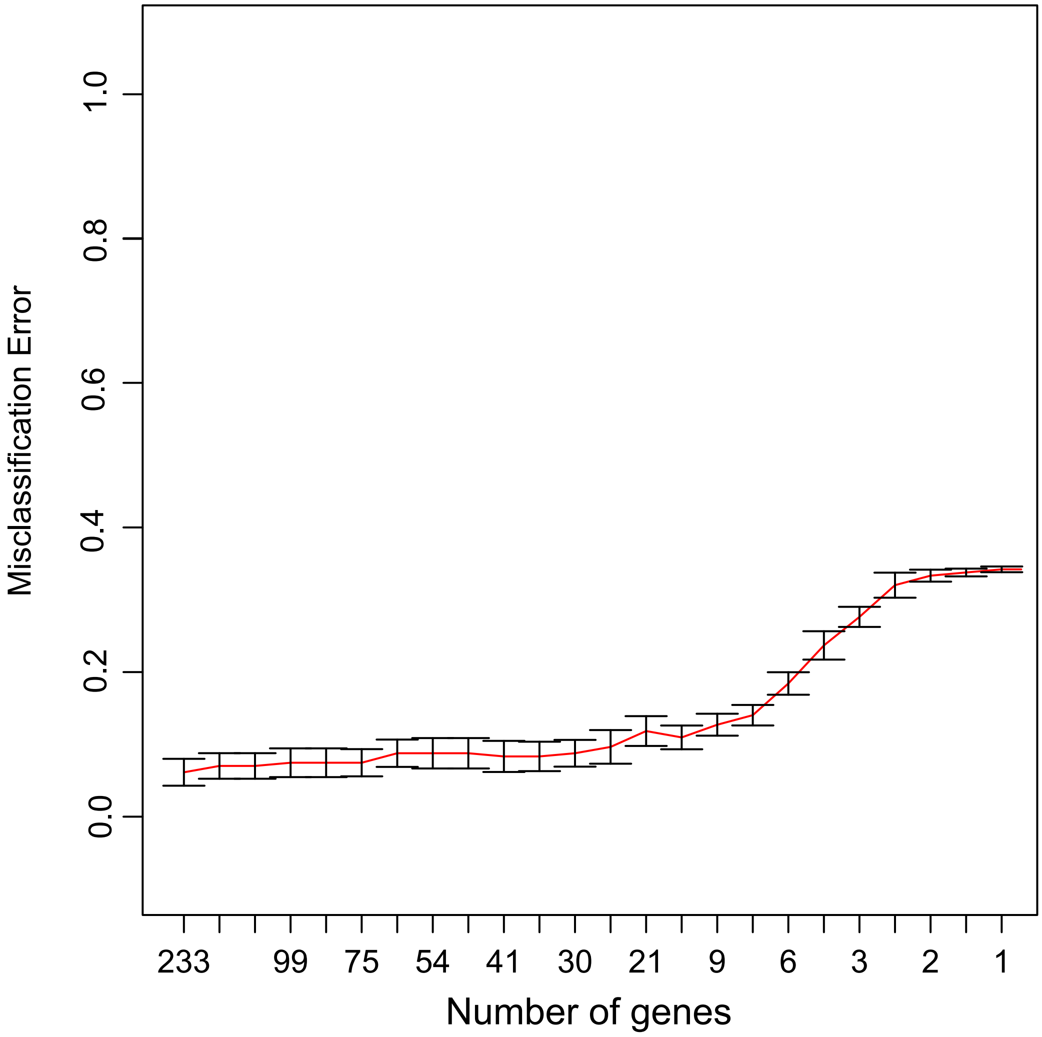

Figure S8. Miscalculation rate of prediction models with given number of genes.

Gene expression data from cohort 3 were used for estimation of minimum number of genes for prediction model. Prediction models with 10 to 20 genes showed acceptable rate of miscalculation (10%).
