# Supplementary material for: Genomic Predictors for Recurrence Patterns of Hepatocellular Carcinoma: Model Derivation and Validation
Source: PLoS Med. 2014 Dec 23;11(12):e1001770. doi: 10.1371/journal.pmed.1001770 (PMC4275163; doi:10.1371/journal.pmed.1001770)
Supplement: Figure S9 — Significance concordance between expression data from microarray experiments and qRT-PCR experiments in 15 genes selected for validation of microarray data. (PDF) [file pmed.1001770.s010.pdf]

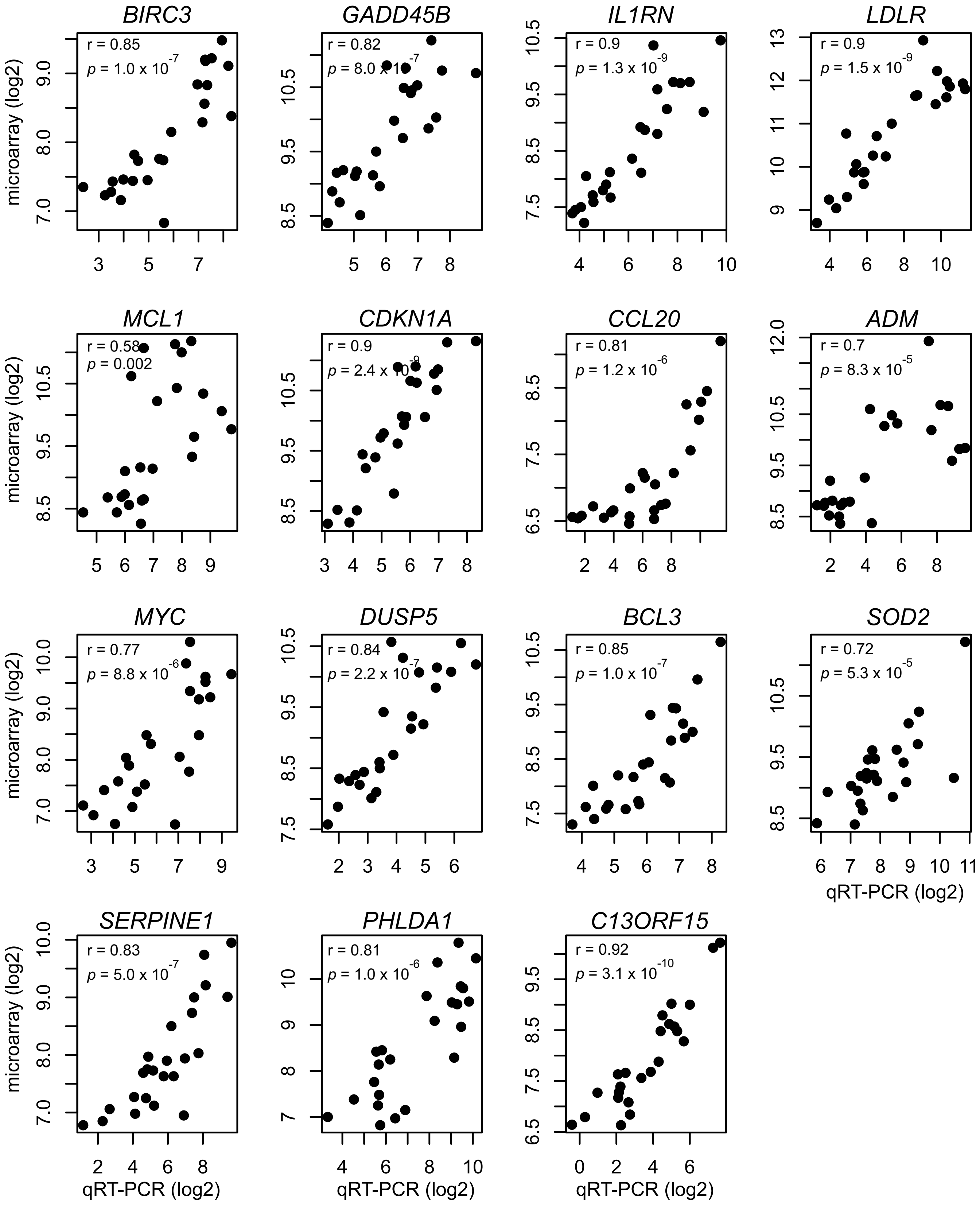

Figure S9. Significance concordance between expression data from microarray experiments and qRT-PCR experiments in 15 genes selected for validation of microarray data.

Scatter plots of normalized gene expression data between two experiments from 24 surrounding tissues from HCC patients. Gene expression data from qRT-PCR experiments were normalized to the expression level of *GAPDH* in the sample.
