# Supplementary material for: Genomic Predictors for Recurrence Patterns of Hepatocellular Carcinoma: Model Derivation and Validation
Source: PLoS Med. 2014 Dec 23;11(12):e1001770. doi: 10.1371/journal.pmed.1001770 (PMC4275163; doi:10.1371/journal.pmed.1001770)
Supplement: Figure S10 — Significance concordance between expression data from microarray experiments and qRT-PCR experiments in four genes selected from multivariate logistic regression analysis. (PDF) [file pmed.1001770.s011.pdf]

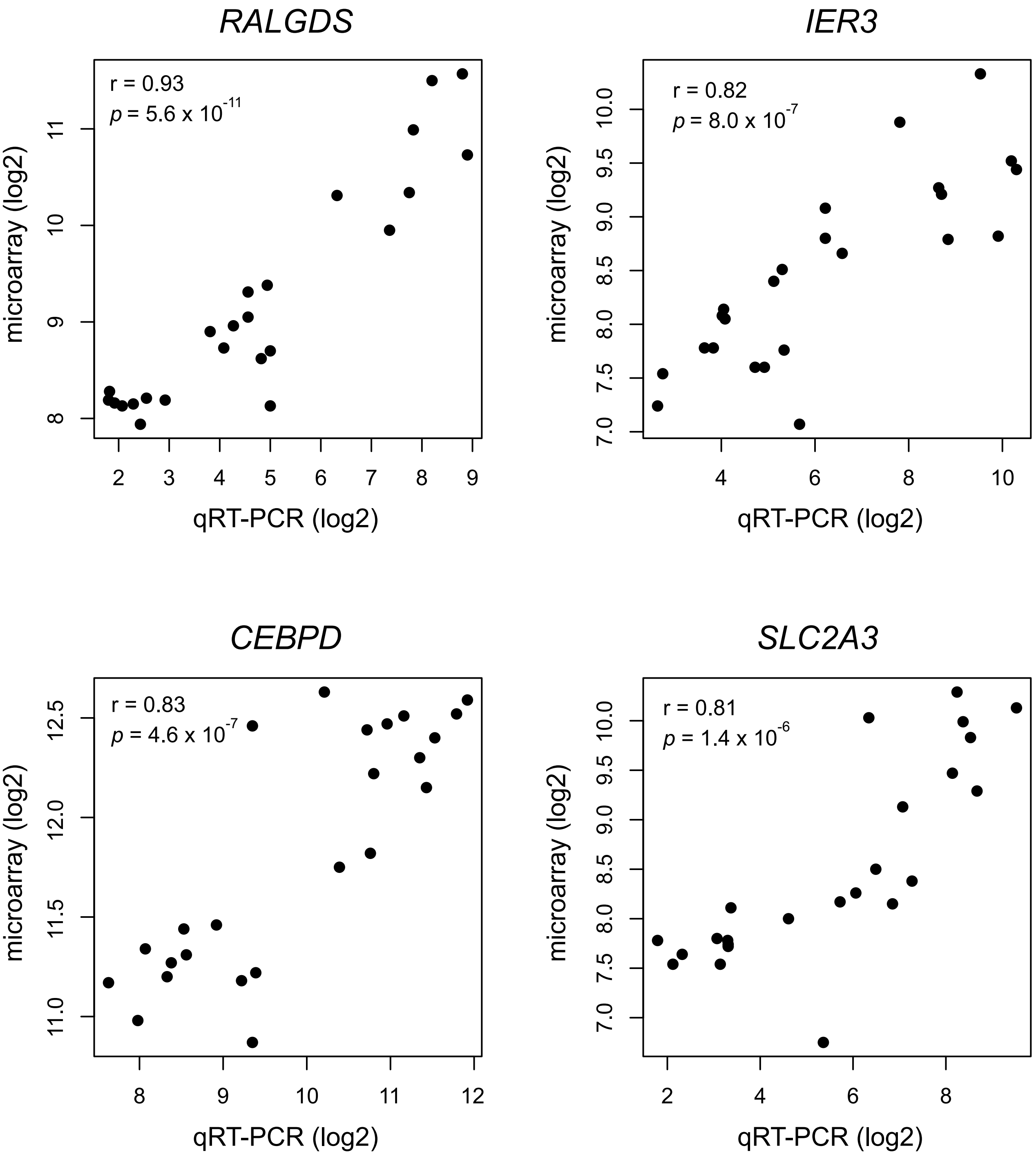


**Figure S10. Significance concordance between expression data from microarray experiments and qRT-PCR experiments in 4 genes selected from multivariate logistic regression analysis.**

Scatter plots of normalized gene expression data between two experiments from 24 surrounding tissues from HCC patients. Gene expression data from qRT-PCR experiments were normalized to the expression level of *GAPDH* in the sample.
