# Supplementary material for: Genomic Predictors for Recurrence Patterns of Hepatocellular Carcinoma: Model Derivation and Validation
Source: PLoS Med. 2014 Dec 23;11(12):e1001770. doi: 10.1371/journal.pmed.1001770 (PMC4275163; doi:10.1371/journal.pmed.1001770)
Supplement: Table S1 — Genes in the HIR signature. (DOCX) [file pmed.1001770.s012.docx]

**Table S1. Genes in Hepatic Injury and Regeneration Signature**

| **Probe ID** | **Gene Symbol** | **Entrez ID** | **Expression Ratios (Log2)** | | |
| --- | --- | --- | --- | --- | --- |
|  |  |  | **PHx** | **DD** | **LD** |
| 227697_at | *SOCS3* | 9021 | 3.66 | 2.96 | 3.24 |
| 202672_s_at | *ATF3* | 467 | 1.79 | 4.59 | 2.52 |
| 36711_at | *MAFF* | 23764 | 2.81 | 2.96 | 2.92 |
| 205476_at | *CCL20* | 6364 | 3.16 | 2.54 | 2.46 |
| 227099_s_at | *LOC387763* | 387763 | 2.22 | 2.63 | 3.14 |
| 222162_s_at | *ADAMTS1* | 9510 | 2.83 | 2.08 | 2.75 |
| 202627_s_at | *SERPINE1* | 5054 | 3.05 | 2.33 | 2.28 |
| 209803_s_at | *PHLDA2* | 7262 | 2.86 | 1.74 | 2.82 |
| 201109_s_at | *THBS1* | 7057 | 2.84 | 2.34 | 2.23 |
| 202628_s_at | *SERPINE1* | 5054 | 2.82 | 2.61 | 1.95 |
| 217996_at | *PHLDA1* | 22822 | 2.78 | 1.98 | 2.59 |
| 218723_s_at | *C13orf15* | 28984 | 3.12 | 2.38 | 1.84 |
| 207526_s_at | *IL1RL1* | 9173 | 2.79 | 1.32 | 3.13 |
| 201466_s_at | *JUN* | 3725 | 1.76 | 3.18 | 1.97 |
| 201110_s_at | *THBS1* | 7057 | 2.38 | 2.28 | 2.21 |
| 217997_at | *PHLDA1* | 22822 | 2.76 | 1.68 | 2.41 |
| 202581_at | *HSPA1B* | 3304 | 1.67 | 3.23 | 1.92 |
| 202431_s_at | *MYC* | 4609 | 2.13 | 2.26 | 2.38 |
| 215078_at | *SOD2* | 6648 | 2.39 | 2.17 | 2.05 |
| 202638_s_at | *ICAM1* | 3383 | 2.08 | 2.12 | 2.36 |
| 212657_s_at | *IL1RN* | 3557 | 1.42 | 2.28 | 2.81 |
| 223467_at | *RASD1* | 51655 | 1.57 | 2.06 | 2.89 |
| 204103_at | *CCL4* | 6351 | 2.04 | 2.75 | 1.68 |
| 201464_x_at | *JUN* | 3725 | 1.54 | 3.19 | 1.65 |
| 200798_x_at | *MCL1* | 4170 | 2.37 | 2.16 | 1.81 |
| 201324_at | *EMP1* | 2012 | 3.25 | 0.96 | 2.12 |
| 229521_at | *FLJ36031* | 168455 | 1.72 | 2.05 | 2.48 |
| 242963_at | *SGMS2* | 166929 | 2.37 | 0.86 | 2.91 |
| 209050_s_at | *RALGDS* | 5900 | 2.21 | 1.76 | 2.10 |
| 226075_at | *SPSB1* | 80176 | 2.69 | 0.97 | 2.17 |
| 209387_s_at | *TM4SF1* | 4071 | 2.78 | 1.06 | 1.95 |
| 209457_at | *DUSP5* | 1847 | 1.46 | 2.50 | 1.81 |
| 243296_at | *NAMPT* | 10135 | 1.84 | 2.00 | 1.93 |
| 221731_x_at | *VCAN* | 1462 | 2.30 | 1.53 | 1.92 |
| 235086_at | *THBS1* | 7057 | 3.16 | 1.29 | 1.30 |
| 215034_s_at | *TM4SF1* | 4071 | 2.55 | 1.19 | 1.97 |
| 202068_s_at | *LDLR* | 3949 | 1.91 | 2.36 | 1.40 |
| 225344_at | *NCOA7* | 135112 | 1.69 | 1.82 | 2.17 |
| 219677_at | *SPSB1* | 80176 | 2.01 | 1.16 | 2.46 |
| 227038_at | *SGMS2* | 166929 | 1.95 | 0.93 | 2.72 |
| 202388_at | *RGS2* | 5997 | 2.45 | 1.79 | 1.34 |
| 201939_at | *PLK2* | 10769 | 1.18 | 2.20 | 2.19 |
| 228846_at | *MXD1* | 4084 | 2.19 | 1.88 | 1.48 |
| 201169_s_at | *BHLHE40* | 8553 | 1.71 | 2.29 | 1.54 |
| 201465_s_at | *JUN* | 3725 | 1.11 | 2.68 | 1.66 |
| 217911_s_at | *BAG3* | 9531 | 1.73 | 1.36 | 2.37 |
| 214056_at | *MCL1* | 4170 | 1.95 | 1.79 | 1.65 |
| 204094_s_at | *TSC22D2* | 9819 | 1.40 | 2.07 | 1.88 |
| 213524_s_at | *G0S2* | 50486 | 1.22 | 1.60 | 2.50 |
| 210845_s_at | *PLAUR* | 5329 | 2.60 | 1.61 | 1.11 |
| 201631_s_at | *IER3* | 8870 | 1.58 | 1.69 | 2.04 |
| 200796_s_at | *MCL1* | 4170 | 1.69 | 1.58 | 2.03 |
| 218810_at | *ZC3H12A* | 80149 | 1.61 | 1.81 | 1.88 |
| 209305_s_at | *GADD45B* | 4616 | 1.47 | 2.14 | 1.68 |
| 213281_at | *JUN* | 3725 | 1.31 | 2.54 | 1.40 |
| 202637_s_at | *ICAM1* | 3383 | 1.86 | 1.60 | 1.77 |
| 202284_s_at | *CDKN1A* | 1026 | 1.48 | 1.44 | 2.30 |
| 242727_at | *ARL5B* | 221079 | 0.94 | 1.73 | 2.46 |
| 200790_at | *ODC1* | 4953 | 1.17 | 0.95 | 2.96 |
| 225612_s_at | *B3GNT5* | 84002 | 2.37 | 1.35 | 1.29 |
| 218000_s_at | *PHLDA1* | 22822 | 1.91 | 1.22 | 1.88 |
| 212856_at | *GRAMD4* | 23151 | 1.47 | 0.93 | 2.57 |
| 233952_s_at | *ZNF295* | 49854 | 1.36 | 1.35 | 2.24 |
| 225557_at | *AXUD1* | 64651 | 1.54 | 2.03 | 1.38 |
| 201170_s_at | *BHLHE40* | 8553 | 1.71 | 2.13 | 1.11 |
| 210056_at | *RND1* | 27289 | 1.31 | 2.06 | 1.56 |
| 209386_at | *TM4SF1* | 4071 | 1.94 | 1.14 | 1.81 |
| 202912_at | *ADM* | 133 | 1.48 | 1.34 | 2.00 |
| *226345_at* | |  | 1.07 | 1.08 | 1.64 |
| 215033_at | *TM4SF1* | 4071 | 1.53 | 1.27 | 1.99 |
| 220088_at | *C5AR1* | 728 | 2.56 | 0.92 | 1.31 |
| 218319_at | *PELI1* | 57162 | 1.69 | 1.08 | 2.00 |
| 212659_s_at | *IL1RN* | 3557 | 0.75 | 1.58 | 2.41 |
| 203499_at | *EPHA2* | 1969 | 1.65 | 1.07 | 2.00 |
| *226498_at* | |  | 1.95 | 1.95 | 0.86 |
| 205119_s_at | *FPR1* | 2357 | 2.31 | 0.91 | 1.46 |
| 205193_at | *MAFF* | 23764 | 2.04 | 1.32 | 1.28 |
| 225262_at | *FOSL2* | 2355 | 1.81 | 1.38 | 1.44 |
| 201739_at | *SGK1* | 6446 | 1.67 | 1.10 | 1.85 |
| 202067_s_at | *LDLR* | 3949 | 1.62 | 1.61 | 1.38 |
| 223394_at | *SERTAD1* | 29950 | 1.48 | 1.39 | 1.72 |
| 238909_at | *S100A10* | 6281 | 1.86 | 1.42 | 1.28 |
| 201920_at | *SLC20A1* | 6574 | 1.24 | 1.59 | 1.74 |
| 202643_s_at | *TNFAIP3* | 7128 | 1.17 | 1.90 | 1.49 |
| 209051_s_at | *RALGDS* | 5900 | 2.06 | 0.84 | 1.64 |
| 201329_s_at | *ETS2* | 2114 | 1.73 | 1.19 | 1.61 |
| 212533_at | *WEE1* | 7465 | 1.42 | 1.88 | 1.18 |
| 201325_s_at | *EMP1* | 2012 | 2.62 | 0.76 | 1.08 |
| 218368_s_at | *TNFRSF12A* | 51330 | 1.65 | 1.17 | 1.64 |
| 202644_s_at | *TNFAIP3* | 7128 | 0.95 | 1.83 | 1.68 |
| 213631_x_at | *DHODH* | 1723 | 1.46 | 0.86 | 2.14 |
| 208152_s_at | *DDX21* | 9188 | 1.65 | 0.83 | 1.98 |
| *232174_at* | |  | 1.48 | 1.48 | 1.10 |
| 208786_s_at | *MAP1LC3B* | 81631 | 1.17 | 1.61 | 1.62 |
| 236725_at | *WWC1* | 23286 | 2.25 | 0.62 | 1.52 |
| 212665_at | *TIPARP* | 25976 | 1.49 | 1.65 | 1.22 |
| 217739_s_at | *NAMPT* | 10135 | 1.85 | 0.81 | 1.68 |
| 226206_at | *MAFK* | 7975 | 1.50 | 1.16 | 1.67 |
| 206359_at | *SOCS3* | 9021 | 1.61 | 1.42 | 1.28 |
| 209723_at | *SERPINB9* | 5272 | 1.61 | 1.09 | 1.57 |
| 207574_s_at | *GADD45B* | 4616 | 1.24 | 1.97 | 1.07 |
| 202014_at | *PPP1R15A* | 23645 | 1.42 | 1.64 | 1.19 |
| 209304_x_at | *GADD45B* | 4616 | 1.21 | 1.90 | 1.13 |
| 221766_s_at | *FAM46A* | 55603 | 1.28 | 1.53 | 1.40 |
| 207630_s_at | *CREM* | 1390 | 1.76 | 1.01 | 1.40 |
| *1565863_at* | |  | 1.94 | 1.94 | 0.99 |
| 210538_s_at | *BIRC3* | 330 | 1.30 | 1.34 | 1.50 |
| 232017_at | *TJP2* | 9414 | 1.81 | 1.18 | 1.14 |
| 228188_at | *FOSL2* | 2355 | 1.21 | 1.42 | 1.49 |
| 222088_s_at | *SLC2A14* | 144195 | 2.24 | 0.89 | 0.98 |
| 210001_s_at | *SOCS1* | 8651 | 1.19 | 1.22 | 1.69 |
| 201108_s_at | *THBS1* | 7057 | 1.57 | 0.96 | 1.56 |
| *244562_s_at* | |  | 1.61 | 1.62 | 1.22 |
| 202497_x_at | *SLC2A3* | 6515 | 1.40 | 1.35 | 1.30 |
| 217738_at | *NAMPT* | 10135 | 1.64 | 0.72 | 1.69 |
| 201473_at | *JUNB* | 3726 | 0.84 | 1.79 | 1.40 |
| 209967_s_at | *CREM* | 1390 | 1.69 | 0.95 | 1.38 |
| 225980_at | *C14orf43* | 91748 | 1.37 | 1.26 | 1.37 |
| 203313_s_at | *TGIF1* | 7050 | 0.92 | 1.56 | 1.52 |
| 205237_at | *FCN1* | 2219 | 1.97 | 0.87 | 1.10 |
| 224654_at | *DDX21* | 9188 | 1.23 | 0.64 | 2.04 |
| 202693_s_at | *STK17A* | 9263 | 1.04 | 0.77 | 2.10 |
| 221215_s_at | *RIPK4* | 54101 | 1.21 | 1.37 | 1.32 |
| 206034_at | *SERPINB8* | 5271 | 1.73 | 0.66 | 1.49 |
| 221485_at | *B4GALT5* | 9334 | 1.62 | 0.71 | 1.54 |
| 212268_at | *SERPINB1* | 1992 | 1.24 | 0.69 | 1.94 |
| 213596_at | *CASP4* | 837 | 1.56 | 0.74 | 1.55 |
| 213006_at | *CEBPD* | 1052 | 1.71 | 0.81 | 1.32 |
| 204011_at | *SPRY2* | 10253 | 1.48 | 1.01 | 1.34 |
| *215206_at* | |  | 1.51 | 1.52 | 1.03 |
| 201531_at | *ZFP36* | 7538 | 1.50 | 1.54 | 0.76 |
| 230511_at | *CREM* | 1390 | 1.94 | 0.60 | 1.24 |
| 210827_s_at | *ELF3* | 1999 | 1.34 | 1.48 | 0.95 |
| 232304_at | *PELI1* | 57162 | 2.12 | 0.72 | 0.90 |
| 204958_at | *PLK3* | 1263 | 1.54 | 1.13 | 1.06 |
| 205895_s_at | *NOLC1* | 9221 | 1.11 | 0.81 | 1.80 |
| *239331_at* | |  | 1.03 | 1.04 | 1.69 |
| 201328_at | *ETS2* | 2114 | 1.34 | 1.16 | 1.19 |
| 212614_at | *ARID5B* | 84159 | 1.51 | 1.07 | 1.09 |
| 1555411_a_at | *CCNL1* | 57018 | 1.09 | 1.46 | 1.12 |
| 202531_at | *IRF1* | 3659 | 1.20 | 1.46 | 1.00 |
| 204908_s_at | *BCL3* | 602 | 1.27 | 0.92 | 1.46 |
| *237310_at* | |  | 1.45 | 1.46 | 0.80 |
| 208892_s_at | *DUSP6* | 1848 | 1.14 | 1.12 | 1.37 |
| 210517_s_at | *AKAP12* | 9590 | 1.43 | 1.18 | 1.00 |
| 203737_s_at | *PPRC1* | 23082 | 1.57 | 0.55 | 1.48 |
| 208893_s_at | *DUSP6* | 1848 | 1.38 | 0.85 | 1.34 |
| 202071_at | *SDC4* | 6385 | 1.35 | 1.05 | 1.14 |
| *1563357_at* | |  | 1.87 | 1.87 | 0.44 |
| 238725_at | *IRF1* | 3659 | 1.34 | 1.08 | 1.07 |
| *242126_at* | |  | 1.71 | 1.72 | 0.66 |
| 228325_at | *KIAA0146* | 23514 | 1.41 | 0.93 | 1.10 |
| 200797_s_at | *MCL1* | 4170 | 1.21 | 1.14 | 1.08 |
| 201859_at | *SRGN* | 5552 | 1.43 | 0.77 | 1.21 |
| 227345_at | *TNFRSF10D* | 8793 | 1.25 | 0.67 | 1.50 |
| 205192_at | *MAP3K14* | 9020 | 1.53 | 0.70 | 1.18 |
| 219343_at | *CDC37L1* | 55664 | 0.94 | 1.00 | 1.47 |
| 209193_at | *PIM1* | 5292 | 1.53 | 0.79 | 1.07 |
| 208891_at | *DUSP6* | 1848 | 1.20 | 0.84 | 1.35 |
| *236495_at* | |  | 1.65 | 1.66 | 0.98 |
| 232213_at | *PELI1* | 57162 | 1.72 | 0.75 | 0.83 |
| 207375_s_at | *IL15RA* | 3601 | 1.25 | 0.74 | 1.30 |
| 225673_at | *MYADM* | 91663 | 1.40 | 0.82 | 1.06 |
| *226497_s_at* | |  | 1.51 | 1.52 | 0.76 |
| 224826_at | *RP5-1022P6.2* | 56261 | 1.30 | 0.62 | 1.33 |
| 217173_s_at | *LDLR* | 3949 | 1.07 | 0.94 | 1.24 |
| *241722_x_at* | |  | 1.13 | 1.14 | 1.03 |
| 224739_at | *PIM3* | 415116 | 1.18 | 1.03 | 0.98 |
| 213146_at | *JMJD3* | 23135 | 1.41 | 0.66 | 1.08 |
| 209034_at | *PNRC1* | 10957 | 1.45 | 0.88 | 0.80 |
| 201596_x_at | *KRT18* | 3875 | 1.14 | 0.66 | 1.32 |
| 222815_at | *RLIM* | 51132 | 1.05 | 0.59 | 1.47 |
| 214508_x_at | *CREM* | 1390 | 1.20 | 0.69 | 1.18 |
| 216236_s_at | *SLC2A14* | 144195 | 1.11 | 0.92 | 1.05 |
| 204420_at | *FOSL1* | 8061 | 1.21 | 0.86 | 0.99 |
| 214805_at | *EIF4A1* | 1973 | 0.89 | 0.69 | 1.44 |
| 209536_s_at | *EHD4* | 30844 | 1.12 | 0.51 | 1.39 |
| 219371_s_at | *KLF2* | 10365 | 0.97 | 1.00 | 1.04 |
| *1564378_a_at* | |  | 1.60 | 1.60 | 0.36 |
| 223096_at | *NOP58* | 51602 | 0.68 | 0.67 | 1.64 |
| 211924_s_at | *PLAUR* | 5329 | 1.41 | 0.69 | 0.85 |
| 220046_s_at | *CCNL1* | 57018 | 0.90 | 1.28 | 0.78 |
| 214326_x_at | *JUND* | 3727 | 1.30 | 0.77 | 0.86 |
| 225955_at | *LOC653506* | 653506 | 1.42 | 0.69 | 0.81 |
| 224761_at | *GNA13* | 10672 | 0.91 | 0.78 | 1.21 |
| 1556067_a_at | *JMJD3* | 23135 | 1.76 | 0.41 | 0.71 |
| 224956_at | *NUFIP2* | 57532 | 1.00 | 0.61 | 1.25 |
| 227175_at | *MCL1* | 4170 | 0.37 | 1.14 | 1.32 |
| 202207_at | *ARL4C* | 10123 | 1.10 | 1.09 | 0.58 |
| 220147_s_at | *FAM60A* | 58516 | 1.11 | 0.75 | 0.91 |
| 227621_at | *WTAP* | 9589 | 0.80 | 0.84 | 1.11 |
| 225604_s_at | *GLIPR2* | 152007 | 1.39 | 0.49 | 0.87 |
| 227534_at | *C9orf21* | 195827 | 0.93 | 0.58 | 1.20 |
| 203140_at | *BCL6* | 604 | 1.14 | 0.76 | 0.80 |
| 213076_at | *ITPKC* | 80271 | 0.91 | 0.51 | 1.26 |
| 213134_x_at | *BTG3* | 10950 | 0.83 | 0.76 | 1.06 |
| 219397_at | *COQ10B* | 80219 | 0.90 | 0.68 | 1.03 |
| 205548_s_at | *BTG3* | 10950 | 1.07 | 0.75 | 0.79 |
| 203973_s_at | *CEBPD* | 1052 | 0.69 | 1.12 | 0.79 |
| 201368_at | *ZFP36L2* | 678 | 0.92 | 0.86 | 0.82 |
| 208785_s_at | *MAP1LC3B2* | 643246 | 0.42 | 0.64 | 1.54 |
| 213560_at | *GADD45B* | 4616 | 0.80 | 1.02 | 0.74 |
| 205419_at | *GPR183* | 1880 | 1.11 | 0.87 | 0.55 |
| 41386_i_at | *JMJD3* | 23135 | 0.97 | 0.76 | 0.78 |
| 211951_at | *NOLC1* | 9221 | 0.69 | 0.40 | 1.38 |
| 215111_s_at | *TSC22D1* | 8848 | 0.82 | 0.90 | 0.73 |
| 225699_at | *C7orf40* | 285958 | 0.47 | 0.50 | 1.45 |
| 200713_s_at | *MAPRE1* | 22919 | 1.02 | 0.46 | 0.89 |
| 215485_s_at | *ICAM1* | 3383 | 0.86 | 0.52 | 0.93 |
| 207667_s_at | *MAP2K3* | 5606 | 0.89 | 0.55 | 0.86 |
| 224927_at | *KIAA1949* | 170954 | 1.02 | 0.56 | 0.70 |
| 218708_at | *NXT1* | 29107 | 0.67 | 0.47 | 1.12 |
| 229630_s_at | *WTAP* | 9589 | 0.59 | 0.72 | 0.95 |
| 212722_s_at | *JMJD6* | 23210 | 0.76 | 0.44 | 1.05 |
| 209545_s_at | *RIPK2* | 8767 | 0.80 | 0.74 | 0.69 |
| 204693_at | *CDC42EP1* | 11135 | 0.81 | 0.54 | 0.88 |
| 202010_s_at | *ZNF410* | 57862 | 0.66 | 0.51 | 1.06 |
| 209747_at | *TGFB3* | 7043 | 0.51 | 0.64 | 1.07 |
| 213459_at | *RPL37A* | 6168 | 0.68 | 0.42 | 1.11 |
| 220990_s_at | *TMEM49* | 81671 | 0.87 | 0.50 | 0.84 |
| 220306_at | *FAM46C* | 54855 | 0.65 | 0.69 | 0.82 |
| *236907_at* | |  | 0.58 | 0.59 | 0.60 |
| 224978_s_at | *USP36* | 57602 | 0.67 | 0.46 | 1.01 |
| 207945_s_at | *CSNK1D* | 1453 | 0.56 | 0.64 | 0.91 |
| 211899_s_at | *TRAF4* | 9618 | 0.47 | 0.43 | 1.11 |
| 218647_s_at | *YRDC* | 79693 | 0.80 | 0.51 | 0.68 |
| 204907_s_at | *BCL3* | 602 | 0.74 | 0.47 | 0.72 |
| 201751_at | *JOSD1* | 9929 | 0.89 | 0.43 | 0.60 |
| 204180_s_at | *ZBTB43* | 23099 | 0.74 | 0.54 | 0.62 |
| 219031_s_at | *NIP7* | 51388 | 0.71 | 0.36 | 0.83 |
| *1570588_at* | |  | 0.69 | 0.70 | 0.45 |
| 219409_at | *SNIP1* | 79753 | 0.67 | 0.46 | 0.74 |
| 202716_at | *PTPN1* | 5770 | 0.81 | 0.53 | 0.52 |
| 204765_at | *ARHGEF5* | 7984 | 0.61 | 0.49 | 0.76 |
| 204181_s_at | *ZBTB43* | 23099 | 0.61 | 0.60 | 0.62 |
| 207643_s_at | *TNFRSF1A* | 7132 | 0.73 | 0.43 | 0.63 |
| 211999_at | *H3F3A* | 3020 | 0.72 | 0.43 | 0.50 |
| 220671_at | *CCRN4L* | 25819 | 0.41 | 0.51 | 0.70 |
| 224097_s_at | *F11R* | 50848 | 0.53 | 0.36 | 0.71 |
| 210954_s_at | *TSC22D2* | 9819 | 0.36 | 0.34 | 0.88 |
| 57703_at | *SENP5* | 205564 | 0.45 | 0.35 | 0.62 |
| 212502_at | *ADO* | 84890 | 0.41 | 0.39 | 0.61 |
| 204436_at | *PLEKHO2* | 80301 | 0.45 | 0.38 | 0.58 |
| 218881_s_at | *FOSL2* | 2355 | 0.56 | 0.26 | 0.51 |
| 225675_at | *C14orf101* | 54916 | -0.42 | -0.31 | -0.43 |
| 201518_at | *CBX1* | 10951 | -0.39 | -0.35 | -0.43 |
| 36612_at | *FAM168A* | 23201 | -0.50 | -0.34 | -0.35 |
| 209688_s_at | *CCDC93* | 54520 | -0.47 | -0.39 | -0.46 |
| 1560042_at | *FAM82A1* | 151393 | -0.79 | -0.24 | -0.33 |
| 202317_s_at | *UBE4B* | 10277 | -0.41 | -0.56 | -0.45 |
| 223440_at | *C16orf70* | 80262 | -0.43 | -0.32 | -0.69 |
| 218596_at | *TBC1D13* | 54662 | -0.53 | -0.34 | -0.60 |
| 223404_s_at | *C1orf25* | 81627 | -0.74 | -0.36 | -0.38 |
| 220770_s_at | *C5orf54* | 63920 | -0.62 | -0.40 | -0.46 |
| 242428_at | *DCUN1D1* | 54165 | -0.65 | -0.57 | -0.27 |
| 1552310_at | *C15orf40* | 123207 | -0.53 | -0.51 | -0.48 |
| 232724_at | *MS4A6A* | 64231 | -0.61 | -0.57 | -0.39 |
| 204020_at | *PURA* | 5813 | -0.43 | -0.54 | -0.61 |
| 202561_at | *TNKS* | 8658 | -0.49 | -0.51 | -0.60 |
| 224847_at | *CDK6* | 1021 | -0.40 | -0.58 | -0.64 |
| 213761_at | *MDM1* | 56890 | -0.69 | -0.58 | -0.37 |
| 209586_s_at | *PRUNE* | 58497 | -0.59 | -0.39 | -0.68 |
| 215731_s_at | *MPHOSPH9* | 10198 | -0.93 | -0.32 | -0.41 |
| 200636_s_at | *PTPRF* | 5792 | -0.54 | -0.61 | -0.51 |
| *226826_at* | |  | -0.80 | -0.81 | -0.40 |
| 235132_at | *LOC254128* | 254128 | -0.77 | -0.40 | -0.50 |
| 44696_at | *TBC1D13* | 54662 | -0.67 | -0.40 | -0.63 |
| 238768_at | *C2orf68* | 388969 | -0.81 | -0.43 | -0.47 |
| *225318_at* | |  | -0.61 | -0.61 | -0.75 |
| 235215_at | *ERCC4* | 2072 | -0.53 | -0.56 | -0.67 |
| 201449_at | *TIA1* | 7072 | -0.66 | -0.58 | -0.52 |
| *243020_at* | |  | -0.89 | -0.89 | -0.33 |
| 227796_at | *ZFP62* | 643836 | -0.82 | -0.46 | -0.56 |
| 224998_at | *CMTM4* | 146223 | -0.42 | -0.84 | -0.60 |
| 238519_at | *RSC1A1* | 6248 | -0.77 | -0.54 | -0.58 |
| 221965_at | *MPHOSPH9* | 10198 | -0.62 | -0.57 | -0.72 |
| 226924_at | *LOC400657* | 400657 | -0.64 | -0.51 | -0.76 |
| 231950_at | *ZNF658* | 26149 | -0.94 | -0.45 | -0.55 |
| 227396_at | *PTPRJ* | 5795 | -0.49 | -0.55 | -0.94 |
| 221740_x_at | *LRRC37A2* | 474170 | -0.64 | -0.71 | -0.62 |
| 209121_x_at | *NR2F2* | 7026 | -0.74 | -0.36 | -0.89 |
| 223184_s_at | *AGPAT3* | 56894 | -0.67 | -0.48 | -0.85 |
| 226148_at | *ZBTB44* | 29068 | -0.39 | -0.71 | -0.91 |
| 228920_at | *ZNF260* | 339324 | -0.73 | -0.65 | -0.66 |
| 226977_at | *C5orf53* | 492311 | -0.56 | -0.79 | -0.70 |
| 1558369_at | *MPHOSPH9* | 10198 | -0.59 | -0.76 | -0.72 |
| 228630_at | *ZNF84* | 7637 | -0.88 | -0.51 | -0.70 |
| 233461_x_at | *ZNF226* | 7769 | -1.02 | -0.47 | -0.65 |
| 223183_at | *AGPAT3* | 56894 | -0.66 | -0.70 | -0.81 |
| 238860_at | *C6orf130* | 221443 | -1.00 | -0.40 | -0.79 |
| 226648_at | *HIF1AN* | 55662 | -0.86 | -0.81 | -0.53 |
| 218820_at | *C14orf132* | 56967 | -0.76 | -0.45 | -1.01 |
| 225332_at | *LOC729082* | 729082 | -0.64 | -0.70 | -0.88 |
| 235306_at | *GIMAP8* | 155038 | -0.73 | -0.60 | -0.89 |
| *225917_at* | |  | -0.78 | -0.79 | -0.49 |
| *231136_at* | |  | -1.26 | -1.27 | -0.47 |
| 217627_at | *ZNF573* | 126231 | -1.18 | -0.55 | -0.55 |
| 223469_at | *PGPEP1* | 54858 | -0.82 | -0.58 | -0.90 |
| *238000_at* | |  | -1.56 | -1.57 | -0.27 |
| *239516_at* | |  | -1.05 | -1.05 | -0.64 |
| 241376_at | *LOC100130097* | 1E+08 | -0.96 | -0.69 | -0.80 |
| 233123_at | *SLC40A1* | 30061 | -1.28 | -0.65 | -0.59 |
| *243039_at* | |  | -1.09 | -1.10 | -0.62 |
| *1562529_s_at* | |  | -1.19 | -1.20 | -0.65 |
| 227909_at | *NCRNA00087* | 644596 | -0.94 | -0.48 | -1.18 |
| 242956_at | *IDH1* | 3417 | -1.62 | -0.45 | -0.54 |
| 210111_s_at | *KLHDC10* | 23008 | -0.67 | -0.67 | -1.28 |
| *239476_at* | |  | -1.48 | -1.49 | -0.50 |
| 231015_at | *KLF15* | 28999 | -0.92 | -0.61 | -1.25 |
| 1553693_s_at | *CBR4* | 84869 | -1.71 | -0.47 | -0.62 |
| 225840_at | *TEF* | 7008 | -0.86 | -0.57 | -1.57 |
| *225221_at* | |  | -1.00 | -1.00 | -0.67 |
| 219648_at | *MREG* | 55686 | -1.16 | -0.71 | -1.24 |
| 219563_at | *C14orf139* | 79686 | -1.16 | -0.90 | -1.11 |
| 1557953_at | *ZKSCAN1* | 7586 | -1.42 | -0.45 | -1.33 |
| *239086_at* | |  | -1.83 | -1.84 | -0.61 |
| 226490_at | *NHSL1* | 57224 | -0.79 | -0.73 | -1.79 |
| *242046_at* | |  | -1.70 | -1.70 | -0.74 |
| 1563160_at | *F11* | 2160 | -2.04 | -0.66 | -0.89 |
| 1560679_at | *LOC151438* | 151438 | -1.65 | -0.56 | -1.49 |
| *1559410_at* | |  | -2.36 | -2.36 | -0.36 |
| 225278_at | *PRKAB2* | 5565 | -1.02 | -0.95 | -1.97 |
| 1554931_at | *CYP4A11* | 1579 | -1.96 | -0.64 | -1.98 |
| 1552569_a_at | *RTP3* | 83597 | -1.34 | -0.93 | -2.72 |
| 214837_at | *ALB* | 213 | -2.50 | -2.99 | -4.14 |

**PHx** denotes Partial Hepatectomy, **DD** deceased donor transplantation, **LD** living donor transplantation.
