# Supplementary material for: Genomic Predictors for Recurrence Patterns of Hepatocellular Carcinoma: Model Derivation and Validation
Source: PLoS Med. 2014 Dec 23;11(12):e1001770. doi: 10.1371/journal.pmed.1001770 (PMC4275163; doi:10.1371/journal.pmed.1001770)
Supplement: Table S2 — Overlap of predicted outcomes by two genomic predictors. (DOCX) [file pmed.1001770.s013.docx]

**Table S2. Overlap of Predicted Outcomes by Two Genomic Predictors**

|  |  | **BROAD signature** | | |
| --- | --- | --- | --- | --- |
|  |  | **High** | **Low** | **Total** |
| **Hepatic Injury Signature** | **HIR** | 54 | 82 | 136 |
|  | **QT** | 39 | 221 | 260 |
|  | **Total** | 93 | 303 | 396 |

**HIR,** Hepatic Injury and Regeneration subgroup, **QT** Quiescent subgroup.

*p* = 7.3 x 10^-8^, χ^2^-test
