# Supplementary material for: Genomic Predictors for Recurrence Patterns of Hepatocellular Carcinoma: Model Derivation and Validation
Source: PLoS Med. 2014 Dec 23;11(12):e1001770. doi: 10.1371/journal.pmed.1001770 (PMC4275163; doi:10.1371/journal.pmed.1001770)
Supplement: Table S3 — Functional categories of HIR signature (DOCX) [file pmed.1001770.s014.docx]

| **Category** | **Number of genes** | **p-Value** |
| --- | --- | --- |
| Cell Death | 93 | 5.52E-23 |
| Cellular Growth and Proliferation | 71 | 6.36E-18 |
| Cancer | 105 | 2.85E-17 |
| Hematological System Development and Function | 51 | 4.58E-17 |
| Tissue Morphology | 51 | 4.58E-17 |
| Hematopoiesis | 45 | 4.87E-15 |
| Inflammatory Response | 59 | 5.61E-14 |
| Cell Cycle | 46 | 8.28E-14 |
| Cellular Development | 64 | 9.69E-14 |
| Cell Morphology | 55 | 1.54E-12 |
| Connective Tissue Disorders | 15 | 8.04E-12 |
| Immunological Disease | 15 | 8.04E-12 |
| Inflammatory Disease | 15 | 8.04E-12 |
| Skeletal and Muscular Disorders | 15 | 8.04E-12 |
| Cellular Movement | 55 | 3.11E-11 |
| Connective Tissue Development and Function | 19 | 4.10E-11 |
| Cellular Function and Maintenance | 29 | 5.05E-11 |
| Gene Expression | 28 | 2.43E-10 |
| Organismal Survival | 52 | 4.16E-10 |
| Tissue Development | 73 | 9.47E-10 |

**Table S3. Functional categories of HIR signature.**
