# Supplementary material for: Genomic Predictors for Recurrence Patterns of Hepatocellular Carcinoma: Model Derivation and Validation
Source: PLoS Med. 2014 Dec 23;11(12):e1001770. doi: 10.1371/journal.pmed.1001770 (PMC4275163; doi:10.1371/journal.pmed.1001770)
Supplement: Table S4 — Transcription factors activated in HIR gene expression signature. (DOCX) [file pmed.1001770.s015.docx]

**Table S4. Transcription Factors Activated in HIR Gene Expression Signature.**

| **Transcription Factor** | **Regulation**  **Z-score** | **P-value*** | **Number of Genes**  **in HIR Signature** |
| --- | --- | --- | --- |
| **NOTCH1** | 2.657 | 6.4 x 10^-5^ | 11 |
| **STAT3** | 2.415 | 1.12 x 10^-11^ | 28 |
| **PDX1** | 2.093 | 1.49 x 10^-4^ | 11 |
| **TP53** | 2.049 | 9.31 x 10^-7^ | 40 |
| **RELA** | 2.034 | 4.83 x 10^-6^ | 18 |

**HIR** denotes Hepatic Injury and regeneration

***** Fisher’s exact test
