# Supplementary material for: Genomic Predictors for Recurrence Patterns of Hepatocellular Carcinoma: Model Derivation and Validation
Source: PLoS Med. 2014 Dec 23;11(12):e1001770. doi: 10.1371/journal.pmed.1001770 (PMC4275163; doi:10.1371/journal.pmed.1001770)
Supplement: Table S5 — Expression and phosphorylation of STAT3 in liver tissues. (DOCX) [file pmed.1001770.s016.docx]

**Table S5. Expression and phosphorylation of STAT3 in liver tissues**

| **Tissues** | **Histoscore of STAT3** | | | **P*** | **Histoscore of pSTAT3** | | | **P*** |
| --- | --- | --- | --- | --- | --- | --- | --- | --- |
|  | **0** | **1** | **2** |  | **0** | **1** | **2** |  |
| **Normal Liver (n=9)** | 0 | 5 | 4 |  | 7 | 1 | 1 |  |
| **ST with Early Recurrence (n = 10)** | 0 | 4 | 6 | 0.57 | 2 | 8 | 0 | 0.001 |
| **ST with Late Recurrence (n = 10)** | 0 | 3 | 7 |  | 4 | 1 | 5 |  |

ST, Surrounding tissue of HCC

*Fisher’s exact test
