# Supplementary material for: Genomic Predictors for Recurrence Patterns of Hepatocellular Carcinoma: Model Derivation and Validation
Source: PLoS Med. 2014 Dec 23;11(12):e1001770. doi: 10.1371/journal.pmed.1001770 (PMC4275163; doi:10.1371/journal.pmed.1001770)
Supplement: Table S6 — Functional categories of genes in the 65-gene risk score. (DOCX) [file pmed.1001770.s017.docx]

| **Category** | **Number of genes** | **p-Value** |
| --- | --- | --- |
| Organismal Development (development of blood vessel) | 11 | 6.61E-04 |
| Cardiovascular Development (development of blood vessel) | 11 | 6.61E-04 |
| Organismal Development (vasculogenesis) | 9 | 4.07E-03 |
| Cardiovascular Development (vasculogenesis) | 9 | 4.07E-03 |
| Cellular Assembly and Organization (cellular protrusion) | 10 | 1.01E-03 |
| Cellular Function and Maintenance (cellular protrusion) | 10 | 1.01E-03 |
| Cell Morphology | 10 | 1.01E-03 |
| Cancer | 29 | 1.24E-03 |
| Gastrointestinal Disease | 29 | 1.24E-03 |
| Organismal Survival | 21 | 8.20E-04 |

**Table S6. Functional categories of genes in RS65 signature.**
