# Supplementary material for: Genomic Predictors for Recurrence Patterns of Hepatocellular Carcinoma: Model Derivation and Validation
Source: PLoS Med. 2014 Dec 23;11(12):e1001770. doi: 10.1371/journal.pmed.1001770 (PMC4275163; doi:10.1371/journal.pmed.1001770)
Supplement: Table S7 — Selected 20 genes from top ten functional categories. (DOCX) [file pmed.1001770.s018.docx]

| **Category** | **Genes** |
| --- | --- |
| Cell Death | *ADM, GADD45B* |
| Cellular Growth and Proliferation | *CDKN1A, SOCS3* |
| Cancer | *BIRC3, FAM64A* |
| Hematological System Development and Function | *IL1RN, MCL1* |
| Tissue Morphology | *LDLR, ZFP36* |
| Hematopoiesis | *DUSP5, ELF3* |
| Inflammatory Response | *IER3, CCL20* |
| Cell Cycle | *EMP1, RSC22D1* |
| Cellular Development | *RALGDS, SOD2* |
| Cell Morphology | *PHLDA1, SERPINE1* |

**Table S7. Selected 20 genes from top 10 functional categories.**
