# Supplementary material for: Genomic Predictors for Recurrence Patterns of Hepatocellular Carcinoma: Model Derivation and Validation
Source: PLoS Med. 2014 Dec 23;11(12):e1001770. doi: 10.1371/journal.pmed.1001770 (PMC4275163; doi:10.1371/journal.pmed.1001770)
Supplement: Table S8 — Concordance between the HIR and HIR20 models. (DOCX) [file pmed.1001770.s019.docx]

**Table S8. Concordance between HIR and HIR20 models**

|  |  | **HIR20 signature** | | |
| --- | --- | --- | --- | --- |
|  |  | **HIR** | **QT** | **Total** |
| **HIR Signature** | **HIR** | 125 | 11 | 136 |
|  | **QT** | 19 | 241 | 260 |
|  | **Total** | 144 | 252 | 396 |

**HIR,** Hepatic Injury and Regeneration subgroup, **QT** Quiescent subgroup.

*p* = 5.0 x 10^-62^, χ^2^-test
