# Supplementary material for: Genomic Predictors for Recurrence Patterns of Hepatocellular Carcinoma: Model Derivation and Validation
Source: PLoS Med. 2014 Dec 23;11(12):e1001770. doi: 10.1371/journal.pmed.1001770 (PMC4275163; doi:10.1371/journal.pmed.1001770)
Supplement: Table S9 — Concordance between the HIR and HIR4 models. (DOCX) [file pmed.1001770.s020.docx]

**Table S9. Concordance between HIR and HIR4 models**

|  |  | **HIR4 signature** | | |
| --- | --- | --- | --- | --- |
|  |  | **HIR** | **QT** | **Total** |
| **HIR Signature** | **HIR** | 103 | 33 | 136 |
|  | **QT** | 30 | 230 | 260 |
|  | **Total** | 133 | 263 | 396 |

**HIR,** Hepatic Injury and Regeneration subgroup, **QT** Quiescent subgroup.

*p* = 9.2 x 10^-38^, χ^2^-test
