# Supplementary material for: Genomic Predictors for Recurrence Patterns of Hepatocellular Carcinoma: Model Derivation and Validation
Source: PLoS Med. 2014 Dec 23;11(12):e1001770. doi: 10.1371/journal.pmed.1001770 (PMC4275163; doi:10.1371/journal.pmed.1001770)
Supplement: Text S1 — Supporting text. (DOCX) [file pmed.1001770.s021.docx]

**SUPPLEMENTARY information**

**Genomic Predictors for Recurrence Patterns of Hepatocellular Carcinoma**

**: Model Derivation and Validation**

**Development of Prediction Model**

In order to generate the probability of hepatic injury for each patient with HCC, expression data from patients who had undergone partial hepatectomy or liver transplantation (training set, Fig 1) were used to build a classifier based on the Bayesian compound covariate predictor (BCCP) algorithm.^1^ Before applying the prognostic classification algorithm, we normalized gene expression data used as training and test sets by centralizing the gene expression level across the tissues. During training of prediction model, the pairing of the samples was not taken into account and prediction model were applied to validation set in blinded manner and clinical significance was assessed later. All samples were considered as independent collection of tissues.

The paragraphs below describe the classifier development algorithms that were applied in the training and test sets.

Let *t_j_* denote the *t* statistic for gene *j* for comparing class 1 to class 2 in the training set:

(1)

where $\bar{x}_{j}^{(m)}$denotes the mean of log expression *j* for training samples in class *m* (*m*=1,2), *s_j_* is an estimate of the intra-class variance for gene *j*, and *n_m_* is the number of cases in the training set in class *m* (*m*=1,2).

Let *x_ij_* denote the log expression for gene *j* in sample *i* of the training set. For each sample of the training set, the compound covariate value is computed as follows:

$C_{i}=\sum_{j\varepsilon Selected} t_{j}x_{ij}$ (2)

Let $\bar{C}^{(1)}$ denote the mean of the *C_i_* values for samples in class 1 in the training set and let $\bar{C}^{(2)}$denote the mean for samples in class 2 of the training set.

The compound covariate for sample *y* in test set is defined by:

$C=\sum_{j\varepsilon Selected} t_{j}y_{j}$ (3)

To classify a case not in the training set with log expression values *Y* = (*y*_1_, *y*_2_, …, *y_p_*), one computes the compound covariate value C using expression (3). The probability that sample *Y* came from class 1 is estimated as:

$\Pr[Y\text{ in class 1]}=\frac{\varphi(C;\bar{C}^{(1)},V)\pi_{1}}{\varphi(C;\bar{C}^{(1)},V)\pi_{1}+\varphi(C;\bar{C}^{(2)},V)(1-\pi_{1})}$ (4)

where $\varphi(C;\bar{C}^{(1)},V)$denotes the Gaussian density of a value *C* when the mean is $\bar{C}^{(1)}$ and the variance is *V*. $\pi_{1}$ denotes the prior probability that the class is 1. The probability that the new sample *Y* is from class 2 is 1 minus the value given in (4). The class with the greater computed probability is predicted for the new sample. The Bayesian compound covariate method was developed by GW Wright et al.^2^

We applied the trained BCCP to the gene expression data of cohort 1 and generated the probability of having hepatic injury signature for each patient. The probability of having hepatic injury signature was significantly associated with disease recurrence (hazard ratio, 3.15; 95% confidential interval, 1.32–7.5; *p* = 0.009) as a continuous variable.

Reference List

1. Radmacher MD, McShane LM, Simon R. A paradigm for class prediction using gene expression profiles. J Comput Biol 2002;9:505-511.

2. Wright G, Tan B, Rosenwald A et al. A gene expression-based method to diagnose clinically distinct subgroups of diffuse large B cell lymphoma. Proc Natl Acad Sci U S A 2003;%19;100:9991-9996.
